# Cognitive Training for Reduction of Delirium in Patients Undergoing Cardiac Surgery: A Randomized Clinical Trial

**DOI:** 10.1001/jamanetworkopen.2024.7361

**Published:** 2024-04-23

**Authors:** Yu Jiang, Yanhu Xie, Panpan Fang, Zixiang Shang, Lihai Chen, Jifang Zhou, Chao Yang, Wenjie Zhu, Xixi Hao, Jianming Ding, Panpan Yin, Zan Wang, Mengyuan Cao, Yu Zhang, Qilian Tan, Dan Cheng, Siyu Kong, Xianfu Lu, Xuesheng Liu, Daniel I. Sessler

**Affiliations:** 1 Department of Anesthesiology, The First Affiliated Hospital of Anhui Medical, University, Hefei, Anhui, China; 2 Department of Anesthesiology, The First Affiliated Hospital of University of Science and Technology of China, Hefei, Anhui, China; 3 Department of Anesthesiology, Nanjing First Hospital, Nanjing Medical University, Nanjing, Jiangsu, China; 4 School of International Pharmaceutical Business, China Pharmaceutical University, Nanjing, Jiangsu, China; 5 Outcomes Research Consortium, Department of Anesthesiology, Cleveland Clinic, Cleveland, Ohio

## Abstract

**IMPORTANCE:**

Postoperative delirium is a common and impactful neuropsychiatric complication in patients undergoing coronary artery bypass grafting surgery. Cognitive training may enhance cognitive reserve, thereby reducing postoperative delirium.

**OBJECTIVE:**

To determine whether preoperative cognitive training reduces the incidence of delirium in patients undergoing coronary artery bypass grafting.

**DESIGN, SETTING, and PARTICIPANTS:**

This prospective, single-blind, randomized clinical trial was conducted at 3 university teaching hospitals in southeastern China with enrollment between April 2022 and May 2023. Eligible participants included those scheduled for elective coronary artery bypass grafting who consented and enrolled at least 10 days before surgery.

**INTERVENTIONS:**

Participating patients were randomly assigned 1:1, stratified by site, to either routine care or cognitive training, which included substantial practice with online tasks designed to enhance cognitive functions including memory, imagination, reasoning, reaction time, attention, and processing speed.

**MAIN OUTCOMES AND MEASURES:**

The primary outcome was occurrence of delirium during postoperative days 1 to 7 or until hospital discharge, diagnosed using the Confusion Assessment Method or the Confusion Assessment Method for Intensive Care Units. Secondary outcomes were postoperative cognitive dysfunction, delirium characteristics, and all-cause mortality within 30 days following the operation.

**RESULTS:**

A total of 218 patients were randomized and 208 (median [IQR] age, 66 [58-70] years; 64 female [30.8%] and 144 male [69.2%]) were included in final analysis, with 102 randomized to cognitive training and 106 randomized to routine care. Of all participants, 95 (45.7%) had only a primary school education and 54 (26.0%) had finished high school. In the cognitive training group, 28 participants (27.5%) developed delirium compared with 46 participants (43.4%) randomized to routine care. Those receiving cognitive training were 57% less likely to develop delirium compared with those receiving routine care (adjusted odds ratio [aOR] 0.43; 95% CI, 0.23-0.77; *P* = .007). Significant differences were observed in the incidence of severe delirium (aOR, 0.46; 95% CI, 0.25-0.82; *P* = .01), median (IQR) duration of delirium (0 [0-1] days for cognitive training vs 0 [0-2] days for routine care; *P* = .008), and median (IQR) number of delirium-positive days (0 [0-1] days for cognitive training vs 0 [0-2] days for routine care; *P* = .007). No other secondary outcomes differed significantly.

**CONCLUSIONS AND RELEVANCE:**

In this randomized trial of 208 patients undergoing coronary artery bypass grafting, preoperative cognitive training reduced the incidence of postoperative delirium. However, our primary analysis was based on fewer than 75 events and should therefore be considered exploratory and a basis for future larger trials.

**Trial Registration:**

Chinese Clinical Trial Registry Identifier: ChiCTR2200058243

## Key Points

**Question:**

Does preoperative cognitive training reduce the incidence of delirium during recovery from coronary artery bypass grafting?

**Findings:**

In a randomized clinical trial that included 208 patients across 3 trial sites, 10 days of in-hospital cognitive training reduced the incidence of postoperative delirium by 57%.

**Meaning:**

These findings suggest that preoperative cognitive training may prevent delirium in patients recovering from coronary artery bypass grafting surgery; however, the results should be considered exploratory and a basis for future larger trials.

## Teaser

This randomized clinical trial assesses whether preoperative cognitive training reduces the incidence of delirium among patients in China undergoing coronary artery bypass grafting surgery.

## Introduction

Postoperative delirium is an acute and transient disruption of cognitive function characterized by impaired attention, cognitive confusion, and altered consciousness. The syndrome remains common in patients recovering from coronary artery bypass grafting (CABG) surgery,^1,2,3^ and it impairs quality of life, imposes a burden on caregivers, and increases health care costs.^4,5,6^ Postoperative delirium is thought to be associated with disturbances in cerebral circulation consequent to cardiopulmonary bypass, sternotomy, embolic load, and hypoperfusion.^7,8^ Microemboli presumably are also associated, even in the absence of overt stroke.^9^

Compromises in attention, short-term memory, and visuospatial processing are associated with postoperative delirium.^10,11^ Cognitive reserve may therefore be a potentially modifiable protective factor that guards against the development of postoperative delirium and postoperative cognitive dysfunction (POCD).^12^ Cognitive reserve mostly develops during childhood and young adult years, but may continue to increase even in old age.^13^

Studies have shown that cognitive reserve is improved by cognitive training in community-dwelling, elderly individuals with benefits lasting months to years.^14,15,16,17^ Cognitive training may, thus, also be helpful in patients undergoing surgery, although the theory remains largely untested.^18^

Patients in our hospitals are typically admitted approximately 1 week before CABG surgery to facilitate medical and presurgical evaluations,^19^ making preoperative cognitive training practical in our setting. We therefore tested the primary hypothesis that preoperative cognitive training reduces the incidence of delirium up to 7 days after CABG surgery while patients remained hospitalized.

## Methods

### Study Design

This multicenter, single-blind, randomized clinical trial was coordinated by the First Affiliated Hospital of Anhui Medical University and approved by its research ethics committee and by the institutional review board at each participating center. This study followed the Consolidated Standards of Reporting Trials (CONSORT) reporting guideline, and participants or their guardians provided written informed consent. Patients were enrolled between April 2022 to May 2023 at 3 university hospitals in southeast China. The trial protocol and statistical plan are available in Supplement 1.

### Participants

We enrolled adults aged 18 years and older at least 10 days before elective CABG surgery. We excluded patients who had a life expectancy of less than 6 months, a history of psychiatric or neurological disorders (including depression, severe central nervous system depression, schizophrenia, epilepsy, and Parkinson or Alzheimer disease), and substantial impairments such as blindness, severe deafness, or dementia that might hinder cognitive testing. We also excluded patients who used psychotropic or opioid medications, had a history of delirium, or a documented history of alcohol abuse or withdrawal within the past 6 months.

### Randomization and Masking

Patients were assigned by computer to either cognitive training or routine care in a 1:1 ratio stratified by study site with random blocking. The computer-generated randomization schema was developed by an unblinded statistician (J.F.Z.) and allocation was concealed from study investigators in sequentially numbered sealed opaque envelopes.

Investigators who provided preoperative cognitive training (C.Y., Q.L.T., and P.P.Y.) opened randomization envelopes immediately after eligibility assessment and formal trial enrollment. Patients were not blinded because they needed to adhere to the protocol. However, surgeons, anesthesiologists, and members of the study team responsible for assessing neurocognitive function (Y.H.X., Y.J., and L.H.C.) and postoperative clinical outcomes were unaware of the patient allocation or treatment.

### Procedures

General anesthesia was induced with etomidate and maintained with sevoflurane in oxygen and air, propofol, fentanyl, and muscle relaxants. Benzodiazepines, ketamine, and dexmedetomidine were avoided to the extent practical, with other aspects of anesthetic management left to the clinicians’ discretion.

Patients randomized to cognitive training were given an internet-connected Android mobile phone with a 6.5-inch diagonal screen. The phones were loaded with a dynamic cognitive exercise mobile application, The Light of Future version 4.13.1 (Beijing Intelligence Technology Co.), that offers a range of online games designed to engage and challenge cognitive abilities including memory, imagination, reasoning, reaction time, attention, and processing speed.^20^ Tasks and their difficulty were initially tailored to patients’ age and educational level with the goal of balancing cognitive stimulation and enjoyment.

Based on previous reports,^21,22,23^ patients were instructed to spend a total of 10 hours on cognitive training. We asked patients to spend at least a full hour per day, over 2 or 3 sessions, and that daily sessions include at least 1 game from each of the 6 available cognitive domains. Details of each participant’s use were recorded by the application, including which games were used, when and for how long the application was used, and participants’ success with each cognitive domain. Unblinded investigators (C.Y., Q.L.T., and P.P.Y.) were readily available to provide in-person support for technical difficulties or study-related concerns. The specific details can be found in the trial protocol in Supplement 1. Participants assigned to routine care were provided with standard hospital attention without any specific cognitive training.

The Montreal Cognitive Assessment (MoCA; range, 0 [worst] to 30 [best]) was used to assess early POCD on the seventh day after surgery or at discharge and to assess cognition at any time point before or after surgery. POCD was defined as a 1-SD decline in MoCA score on postoperative day 7 or at discharge (if earlier) compared with baseline. This definition aligns with previous definitions of POCD and current recommendations for defining POCD or delayed neurocognitive recovery.^24,25^ Additionally, the Revised Telephone Interview for Cognitive Status (TICS-m; range, 0 [worst] to 50 [best]) was used to evaluate cognition 1 month after surgery.

### Outcomes

Our primary outcome was occurrence of delirium during postoperative days 1 to 7 while patients remained hospitalized, assessed using the confusion assessment method (CAM)^26^ or CAM for the intensive care unit (CAM-ICU),^27^ as appropriate. Starting the day after surgery, evaluations were conducted twice daily for 7 consecutive postoperative days, specifically from 8:00 AM to 10:00 AM and from 6:00 PM to 8:00 PM. Comprehensive reviews of progress notes, nursing documents, and medical records were conducted. Delirium was defined as any positive result on aforementioned methods, independent of the number of positive assessments. Severe delirium was evaluated with the Memorial Delirium Assessment Scale. Outcomes were assessed by investigators specifically trained in recognition and assessment of delirium (Y.H.X., Y.J., and L.H.C.).

Secondary outcomes included occurrence of POCD on postoperative day 7 or at discharge (if earlier), cognition 1 month after surgery, 30-day all-cause mortality, durations of ICU and hospital care, duration of delirium (defined as the period between the first and last delirium-positive day, even if there were nondelirious days in between), and the total number of delirium-positive days in patients who developed delirium.

### Statistical Analysis

In a previous study of patients recovering from CABG, the incidence of postoperative delirium was 35%.^28^ We powered our trial to detect a 50% relative reduction in any occurrence of delirium from a 35% baseline consequent to cognitive training. A total of 96 patients were required in each group for a 2-tailed α = .05 with 80% power. Assuming a potential dropout rate of 10%, we planned to enroll a total of 214 patients (107 in each group).

Continuous variables are reported as means (SDs) or medians (IQRs) depending on their distribution as assessed by Kolmogorov-Smirnov test. Categorical variables are presented as frequencies and percentages. Between-group comparisons were performed using the independent sample *t*, Mann-Whitney *U*, χ^2^, or Yates continuity-corrected χ^2^ tests, as appropriate. Baseline characteristics in each group were assessed with absolute standardized differences, defined as the absolute difference in means or proportions divided by the pooled SD. Baseline characteristics were considered imbalanced between groups with absolute standardized differences exceeding the following calculation,^29^ where *n*1 represents the sample size of the cognitive training group and *n*2 represents the sample size of the routine care group:


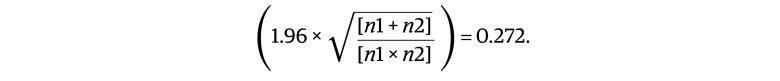
.

Patients who engaged in cognitive training for 3 or more hours were considered to have met our minimum compliance threshold. We used modified intention-to-treat analysis, which was defined as patients who were primarily analyzed within the groups to which they were assigned, whether the minimum compliance threshold of training was met, excluding those without any record of follow-up or canceled surgical procedures. Additionally, a sensitivity analysis (per-protocol population) was conducted after excluding 6 patients who did not meet our minimum compliance definition from the patients assigned to cognitive training. Analysis investigating the effects of cognitive training on the incidence of delirium was conducted using logistic regression techniques, and analyses of delirium duration and delirium-positive days were conducted using the zero-inflated Poisson regression model. Regarding the assessment of delirium severity, an ordinal logistic regression model was used to model no delirium, delirium, and severe delirium. Furthermore, a post hoc analysis incorporating study site, age, surgical technique, baseline educational level, and cognitive function of the participants was conducted.

Predefined subgroup analyses were performed based on surgical methods, age, sex, education level, and baseline cognitive function. Time-to-event results were analyzed using Kaplan-Meier survival analyses, and differences between groups were examined with log-rank tests. Cuzick tests assessed a potential dose-response association of the total hours of cognitive training with incidence of delirium. Imputation was employed to analyze the missing data. Statistical analysis was performed using RStudio version 4.1.0 (Posit PBC). Tests were 2-sided, with *P* values < .05 considered statistically significant for all analyses.

## Results

### Participants

**Figure 1.**
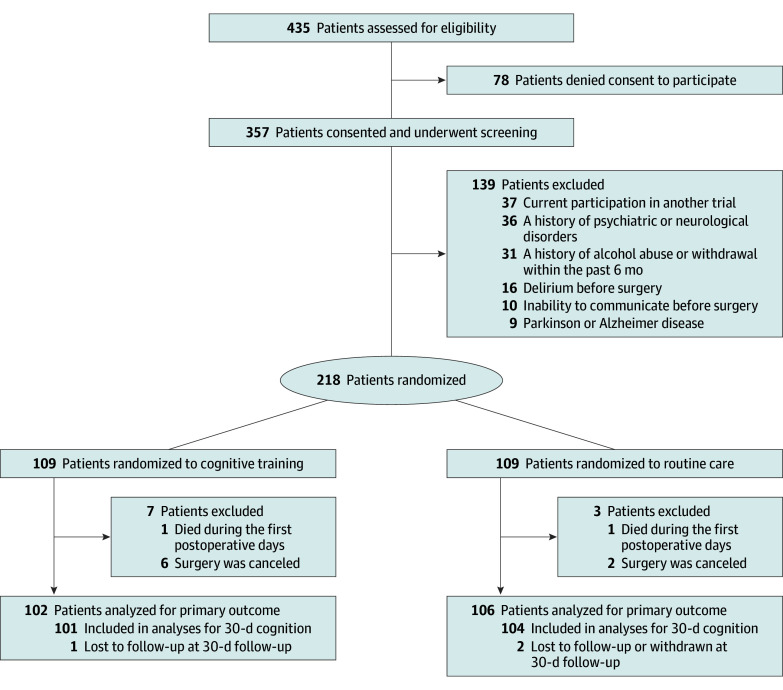
Diagram of Study Participant Flow

Among 435 patients initially screened, 218 met eligibility criteria, consented, and were randomly assigned (see eTable 1 in Supplement 2 for information on recruitment by center). Among the enrolled patients, 8 were excluded because surgery was canceled, and 2 died during the first postoperative days without any record of follow-up. Major outcome data were available for 102 patients assigned to cognitive training and 106 patients assigned to routine care. Of the 208 total participants (median [IQR] age, 66 [58-70] years; 64 female [30.8%]; 144 male [69.2%]), 3 (1.4%) were lost to follow-up by postoperative day 30 (Figure 1). Of all participants, 95 (45.7%) had only a primary school education, and 54 (26.0%) had finished high school. Of all patients, 187 (90.0%) underwent off-pump CABG including 91 in the cognitive training group (89.2%) and 96 in the routine care group (90.6%). Of all participants, 84 (40.4%) had cognitive impairment upon enrollment. Of the 102 patients in the cognitive training group, 39 (38.2%) had mild cognitive impairment, while 45 of the 106 patients in the routine care group (42.5%) had mild cognitive impairment. Baseline characteristics and intraoperative variables were comparable in each treatment group (Table 1 and eTable 2 in Supplement 2). Furthermore, there were no baseline differences observed in patients who received less cognitive training (≤5 hours) and those who received more (>5 hours) (eTable 3 in Supplement 2).

**Table 1.** Characteristics of Patients at Baseline

Characteristic	Participants, No. (%) (N = 208)		Absolute standardized difference
	Cognitive training (n = 102)	Routine care (n = 106)	
Sex			
Female	25 (24.5)	39 (36.8)	0.269
Male	77 (75.5)	67 (63.2)	
Age, median (IQR), y	65 (58-70)	66 (58-70)	0.047
American Society of Anesthesiologists physical status level			
I-II	3 (2.9)	1 (0.9)	0.145
III	76 (74.5)	81 (76.4)	
IV	23 (22.5)	24 (22.6)	
Height, median (IQR), cm	166 (162-170)	165 (160-170)	0.154
Weight, median (IQR), kg	65 (59-77)	65 (58-70)	0.194
Body mass index, mean (SD)^a^	25 (4)	24 (3)	0.114
Education level			
Primary school or below	43 (42.2)	52 (49.1)	0.168
Middle school	29 (28.4)	30 (28.3)	
High school or above	30 (29.4)	24 (22.6)	
Charlson comorbidity index, median (IQR)	1 (0-2)	1 (1-2)	0.140
Age-adjusted Charlson comorbidity index, median (IQR)	3 (2-4)	3 (2- 4)	0.057
Montreal Cognitive Assessment, median (IQR)	26 (25-28)	26 (24-28)	0.167
Frailty score, median (IQR)	1 (0-2)	1 (0-2)	0.037
Mild cognitive impairment^b^	39 (38.2)	45 (42.5)	0.086
Geriatric Depression Scale, median (IQR)	0 (0-1)	0 (0-1)	0.027
Comorbidities			
Hypertension	71 (69.6)	68 (64.2)	0.116
Diabetes	33 (32.4)	36 (34.0)	0.034
Cerebral infarction	41 (40.2)	36 (34.0)	0.129
Transient ischemic attack	18 (17.6)	17 (16.0)	0.043
Moderate or severe liver disease	10 (9.8)	11 (10.4)	0.019
Sample size in each study site^c^			
Site 1	22 (21.6)	29 (27.4)	NA
Site 2	69 (67.6)	68 (64.1)	
Site 3	11 (10.8)	9 (8.5)	

Abbreviation: NA, not applicable.

^a^ Body mass index was calculated as weight in kilograms divided by height in meters squared.

^b^ Mild cognitive impairment was defined by a Montreal Cognitive Assessment score less than 26 (plus 1 point if <12 years of education).

^c^ Site 1, represents the First Affiliated Hospital of Anhui Medical University; site 2, the First Affiliated Hospital of University of Science and Technology of China; and site 3, the Nanjing First Hospital affiliated with Nanjing Medical University.

### Exposure to Cognitive Training

Among the 102 patients assigned to cognitive training, 6 (5.9%) reported participating in preoperative cognitive training for less than 3 hours. Specifically, 2 patients completed only a single hour of training, while the remaining 4 patients engaged for only 2 hours. Overall, 96 of 102 patients (94.1%) met our 3-hour minimum compliance criteria. The duration of preoperative cognitive training varied widely, ranging from 1 to 9 hours, with a median (IQR) time of 6 (5-7) hours (eFigure 1 in Supplement 2).

### Primary Outcomes

During the initial 1 to 7 days after surgery, 74 of 208 participants (35.6%) experienced delirium. Overall, 28 of the 102 patients randomized to cognitive training (27.5%) developed delirium whereas 46 of the 106 patients assigned to routine care (43.4%) experienced delirium. Adjusting for center effects, patients receiving cognitive training were less likely to develop delirium than those receiving routine care (adjusted odds ratio [aOR], 0.42; 95% CI, 0.23-0.77; *P* = .006) (Table 2). Adjusting for center, age, surgical technique, baseline educational level and cognitive function, patients receiving cognitive training were 57% less likely to develop delirium than those receiving routine care (aOR, 0.43; 95% CI, 0.23-0.77; *P* = .007) (eTable 4 in Supplement 2). In a per-protocol analysis, we excluded the 6 patients who did not meet our minimum compliance definition from the patients assigned to cognitive training, which yielded similar results for the major outcome (aOR, 0.38; 95% CI, 0.20-0.71; *P* = .003) (eTable 5 in Supplement 2).

**Table 2.** Postoperative Delirium Characteristics

Characteristic	Participants, No. (%) (N = 208)		Odds ratio (95% CI)		*P* value adjusted for center
	Cognitive training (n = 102)	Routine care (n = 106)	Unadjusted	Adjusted for center	
Primary outcome: postoperative delirium^a^	28 (27.5)	46 (43.4)	0.49 (0.27-0.88)	0.42 (0.23-0.77)	.006
Secondary outcome					
Postoperative delirium onset, d					
0 to 1	13 (12.7)	22 (20.8)	0.56 (0.26-1.16)	0.50 (0.22-1.06)	.08
0 to 2	27 (26.5)	41 (38.7)	0.57 (0.31-1.02)	0.50 (0.27-0.93)	.03
0 to 7	28 (27.5)	46 (43.4)	0.49 (0.27-0.88)	0.42 (0.23-0.77)	.006
Severe delirium^b^	13 (12.7)	17 (16.0)	0.53 (0.30-0.94)	0.46 (0.25-0.82)	.01
Delirium duration, median (IQR), d^c^	0 (0-1)	0 (0-2)	NA	NA	.008
No. of delirium-positive days, median (IQR)^c^	0 (0-1)	0 (0-2)	NA	NA	.007

Abbreviation: NA, not applicable.

^a^ Analysis of the primary outcome was conducted using a logistic regression model.

^b^ An ordinal logistic regression model was used to model no delirium, delirium, and severe delirium, simultaneously on all patients.

^c^ Analyses of delirium duration and delirium-positive days were conducted using the zero-inflated Poisson regression model.

### Secondary Outcomes

Time-to-event analysis revealed distinct differences in the cumulative incidence of delirium in patients assigned to cognitive training vs routine care (hazard ratio, 0.60; 95% CI, 0.38-0.94; log-rank *P* = .01) (eFigure 2 in Supplement 2). Significant differences were observed in the incidence of severe delirium (aOR, 0.46; 95% CI, 0.25-0.82; *P* = .01), median (IQR) duration of delirium (0 [0-1] days for cognitive training vs 0 [0-2] days for routine care; *P* = .008), median (IQR) number of delirium-positive days (0 [0-1] days for cognitive training vs 0 [0-2] days for routine care; *P* = .007) (Table 2 and eFigure 3 in Supplement 2). Moreover, none of the other secondary outcomes differed significantly, including the incidence of POCD on day 7 after surgery or on the day of discharge, cognitive function assessed by MoCA or TICS-m at any time points (baseline, preoperative, discharge, and 1 month after surgery), 30-day all-cause mortality, or durations of ICU and hospital care (Table 3 and eFigure 4 in Supplement 2).

**Table 3.** Secondary Outcomes

Characteristic	Participants, No. (%) (N = 208)		*P* value
	Cognitive training (n = 102)	Routine care (n = 106)	
Discharge Montreal Cognitive Assessment score, median (IQR)	24 (24-25)	25 (24-26)	.09
Postoperative Cognitive Dysfunction score, median (IQR)^a^	56 (54.9)	49 (46.2)	.21
Telephone interview for cognitive status score, median (IQR)	34 (32-36)	34 (32-35)	.06
ICU length of stay, median (IQR), d^b^	1 (1-1)	1 (1-1)	.40
ICU readmission within 30 d	5 (4.9)	5 (4.7)	.95
Postoperative length of stay, median (IQR), d	11 (8-12)	10 (9-11)	.28
All-cause mortality within 30 d	0 (0)	0 (0)	NA
Postoperative intubation time, median (IQR), h^c^	3 (2-3)	3 (2-3)	.11
No. of days alive and out of the hospital within 30 d, median (IQR), d	19 (18-22)	20 (19-21)	.20

Abbreviations: ICU, intensive care unit; NA, not applicable.

^a^ Postoperative cognitive dysfunction was defined as a 1 SD decrease from baseline in Montreal Cognitive Assessment score at postoperative day 7 or discharge.

^b^ Number of days in the ICU from the end of operation to discharge.

^c^ Duration from the end of the procedure to extubation.

### Exploratory Analysis

**Figure 2.**
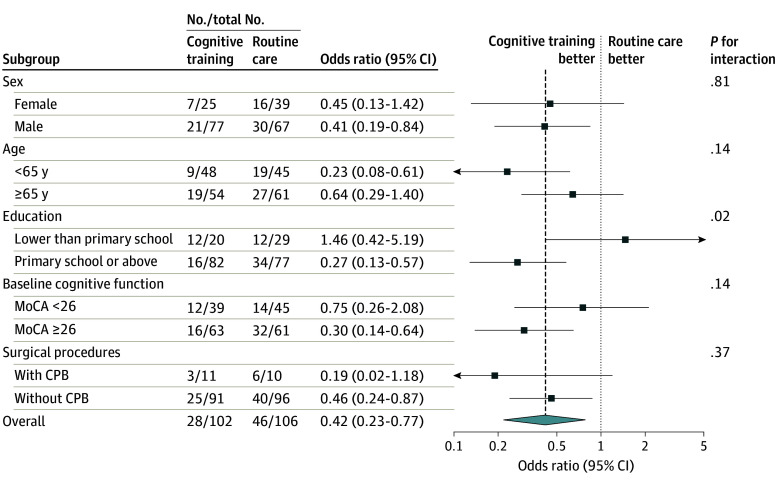
Subgroup Analysis of Primary Outcome *P* values for interaction between subgroup and cognitive training are presented. CPB indicates cardiopulmonary bypass; MoCA, Montreal Cognitive Assessment.

In a predefined subgroup analysis, we compared the effect of cognitive training on subgroups with less or more than primary school education. Cognitive training was effective for preventing delirium in patients with more education (OR, 0.27; 95% CI, 0.13-0.57), whereas there was no significant benefit in those with less education (OR, 1.46; 95% CI, 0.42-5.19; *P *for interaction = .02). No significant interactions were observed on sex, age, operation methods, or baseline cognitive function (Figure 2). The risk of delirium was inversely associated with the amount of cognitive training (eTable 6 and eFigure 5 in Supplement 2).

## Discussion

Evidence suggests that cognitive activities may improve cognition in older adults by inducing structural changes in the brain.^17,30,31^ This randomized clinical trial provides limited evidence suggesting that preoperative cognitive training may also prevent delirium in patients recovering from CABG surgery. Furthermore, benefit was apparent despite a median training period lasting only 6 of 10 requested hours.

As in most perioperative delirium trials, delirium occurred on postoperative days 1 to 3.^32,33^ The incidence of delirium after CABG surgery is reported to be 12% to 50%,^11,28,34,35^ with more recent reports generally being lower. The risk of delirium increases markedly in patients older than 65 years.^14,36^ Our patients had a median age of 66 years, meaning that one-half were in the age range where delirium is most common.

Another baseline factor to consider is educational level, which is inversely associated with delirium and POCD.^3^ The overall educational level of elderly Chinese patients is low,^37^ which was also reflected in our participants, with 45.7% having only a primary school education, and only 26.0% having finished high school. Along those lines, cognitive training was most effective in patients who had finished primary school, although the interaction may be spurious. Finally, people with baseline cognitive impairment are especially vulnerable to postoperative delirium,^38^ and 40.4% of our patients were impaired upon enrollment. Given the age and educational level of our patients, along with their baseline cognition, the observed 43.4% incidence of delirium in our reference patients seems reasonable.

The Prevention of Early Postoperative Decline trial,^18^ a feasibility study of 40 patients undergoing cardiac surgery, suggested that patients scheduled for cardiac surgery were more likely to comply with cognitive training in the preoperative stage, compared with other stages of the perioperative period. Another in-hospital cognitive training trial,^39^ which provided consistent supervision along with allotted breaks to prevent stress and help consolidate learning, provided some meaningful gains in cognitive function but was a pilot investigation that trained just 50 patients. Finally, a recent randomized clinical trial^40^ reported that preoperative cognitive training reduced delirium by 42% in 125 older patients recovering from major noncardiac surgery; however, the conclusion was derived from a post hoc analysis that removed 4 patients who did not meet the minimum adherence criteria. A 42% benefit from outpatient cognitive training is slightly smaller than the 57% reduction we observed, suggesting—unsurprisingly—that in-hospital cognitive training might be more effective than home training. We note, though, that small trials often overestimate treatment effect size and that the true benefit (if any) is probably considerably smaller than we observed.

Our cognitive training was conducted in hospitals because patients scheduled for CABG surgery in China generally have a median hospital stay of 9 days before surgery to complete all the requisite examinations and presurgical preparations.^19^ Even in our patients who were hospitalized and personally encouraged to comply, the median cognitive training duration was only 6 of the recommended 10 hours which, nonetheless, surpassed the cumulative duration noted in prior trials.^18,40^ Future cognitive training research should identify better methods to encourage patient adherence, which may in turn enhance benefit.

As might be expected, exploratory analysis hinted at an improved benefit from increased training time. However, there were only 28 patients assigned to cognitive training who developed delirium, giving us little power to evaluate the association of training duration with delirium prevention. Furthermore, training duration was presumably nonrandom. Specifically, it is likely that patients with better cognitive function complied better. Less delirium in patients who trained more may, thus, represent a confounder rather than an association.

### Limitations

This study has some limitations. Participants randomized to cognitive training were, of course, aware of their treatments and interacted with unblinded trial personnel. Therefore, some apparent benefit of cognitive training may have actually resulted from increased social interaction with nurses and trial personnel rather than from the cognitive exercises.^41^ Comprehensive cognitive function assessments across domains were not conducted, and the underlying mechanism for training effectiveness in preventing delirium remains unclear.

Whether off-pump CABG reduces postoperative neurologic dysfunction remains unclear.^42^ While our subgroup analysis showed no significant interaction between the surgical approaches and the intervention, 90% of patients had off-pump CABG, which provided little power for distinguishing between the methods.

## Conclusions

The results of this randomized clinical trial suggest that preoperative cognitive training may reduce delirium in patients recovering from CABG surgery. Furthermore, substantial benefit was apparent across considerable variation in total training times. However, our primary analysis was based on less than 75 events and, thus, was fragile. Moreover, our intervention was implemented during the preoperative hospitalization period, which is an unusual approach that presumably enhanced training intensity. Less benefit is therefore likely in patients receiving outpatient care. Our results should, thus, be considered exploratory and a basis for future larger trials.

## Notes

Supplement 1.
Trial Protocol and Statistical Plan

Supplement 2. **eTable 1.** Recruitment by Centre **eTable 2.** Intraoperative Measures and Surgical Characteristics **eTable 3.** Baseline Characteristics of Patients by Cognitive Training Time **eFigure 1**. The Light of Future Gameplay Distribution. **eTable 4.** Post-Hoc Analysis Incorporating Baseline Variables **eTable 5.** Per-Protocol Analysis of Primary Outcomes **eFigure 2.** Cumulative Incidence of Delirium in Patients Randomized to Routine Care (Blue) and Cognitive Training (Red) **eFigure 3.** Postoperative Delirium Onset and Duration **eFigure 4.** MoCA Scores Over Time **eTable 6.** Potential Association Between the Total Hours of Cognitive Training and the Risk of Delirium **eFigure 5.** Association of the Duration of Cognitive Training and the Risk of Delirium

Supplement 3.
Nonauthor Collaborators

Supplement 4.
Data Sharing Statement
