# Supplementary material for: Cognitive Training for Reduction of Delirium in Patients Undergoing Cardiac Surgery: A Randomized Clinical Trial
Source: JAMA Netw Open. 2024 Apr 23;7(4):e247361. doi: 10.1001/jamanetworkopen.2024.7361 (PMC11040409; doi:10.1001/jamanetworkopen.2024.7361)
Supplement: Supplement 1. — Trial Protocol and Statistical Plan [file jamanetwopen-e247361-s001.pdf]

(CT-LIFE trail)

PROTOCOL

Research name: a multicenter randomized controlled trial for the effect of preoperative cognitive training on incidence of postoperative delirium in patients undergoing coronary artery bypass grafting surgery

Abbreviations: CT-LIFE-Delirium

Version number: 1.2

Stage: Final

Trial Registration

Chinese Clinical Trial Registry (ChiCTR2200058243)

Contact Names and Details

Leader sponsor

The First Affiliated Hospital of Anhui Medical University

Participating centers:

(1) The First Affiliated Hospital of Anhui Medical University.

(2) The First Affiliated Hospital of University of Science and Technology of China

(3) Nanjing First Hospital Affiliated to Nanjing Medical University

Chief investigator: Xuesheng Liu, MD, Xianfu Lu, M.D.

Trial Statistician: Jifang Zhou

Trial Steering Committee: Lihai Chen, Xuesheng Liu, Qiying Shen, Lili Tang

Data Monitoring and Ethics Committee: Yinguang Fan, Jiamei Liu, Qingfeng Wei

Programme summary

Name of study	Preoperative cognitive training for reduction of delirium in patients recovering from coronary artery bypass grafting (CT-LIFE): a multicenter, single-blinded, randomized trial
Purpose of study	To evaluate the effectiveness of preoperative cognitive training in reducing postoperative delirium incidence among patients undergoing coronary artery bypass grafting.
Research Design	Prospective, multicenter, large sample, randomized controlled study design
Research population	Adults aged ≥18 years who were scheduled for elective coronary artery bypass grafting with general anaesthesia
Selection criteria	(1) Sign the informed consent；<br>(2) Patients who plan to undergo isolated coronary artery bypass grafting with general anaesthesia；<br>(3) Adults aged ≥18 years；<br>(4) Enrolled participants at least 10 days prior to their surgical procedure.
Exclusion criteria	(1) Patients who had a life expectancy <6 months;<br>(2) A history of psychiatric or neurological disorders (including depression, severe central nervous system depression, schizophrenia, epilepsy, and Parkinson's or Alzheimer's disease);<br>(3) Significant impairments such as blindness, severe deafness, or dementia that might hinder cognitive testing;<br>(4) Prior use of psychotropic or opioid medications, a previous history of delirium before surgery;<br>(5) A documented history of alcohol abuse or withdrawal within the past six months;<br>(6) Inability to communicate effectively before surgery due to coma or dementia;<br>(7) Ongoing participation in another clinical trial;<br>(8) Planned re-operation within seven days of the first.
Research Groups	(1) Cognitive training group<br>(2) Routine care group
Main indicators	The incidence of delirium from postoperative day 1 to postoperative day 7 or before discharge, whichever occurred first.
Secondary indicators	(1) cumulative incidence of POD;<br>(2) incidence of severe delirium;<br>(3) postoperative cognitive dysfunction (POCD) on postoperative day 7 or at discharge if earlier;<br>(4) cognitive function one month after surgery;<br>(5) 30-day all-cause mortality;<br>(6) durations of intensive care unit and postoperative hospital care;<br>(7) elapsed time from the end of surgery to delirium onset;

	(8) duration of delirium;<br>(9) the total number of delirium-positive days;<br>(10) cognitive function at three time points;<br>(11) postoperative intubation time;<br>(12) intensive care unit readmission within 30 days after surgery.<br>(13) number of days alive and out of the hospital within 30 days.
Statistical analysis	Comparison of incidence of delirium, characteristics of delirium, and prognosis of patients
Expected test progress	One year

Table of Contents

1.Background 5
2.Objective 7
2.1Primary Aim 7
2.2 Secondary Aims 7
3.1 Investigational Plan 8
3.2 Inclusion /Exclusion/Drop criteria 8
3.2.1 Inclusion criteria 8
3.2.2 Exclusion criteria 8
3.2.3 Drop criteria 9
3.3 Research device and procedures 9
3.3.1 Research device 9
3.3.2 Cognitive training group 9
3.3.3 Routine care group 10
3.4 Randomization Procedure 10
3.5 Blinding 11
3.6 Anesthesia protocol 11
3.6.1 Preoperative management 11
3.6.2 Protocol of anesthesia 11
3.6.3 Airway management 11
3.7. Outcome measures 11
3.7.1 Preoperative measures 11
3.7.2 Intraoperative measures 12
3.7.3 Postoperative outcomes 12
4. Statistical Analysis Plan 13
4.1 Sample size calculation 13
4.2 Statistical methods 14
4.2.1 Data set 14
4.2.2 Data presentation 14
4.2.3 General considerations 14
4.2.4 Analysis of primary endpoint 15
4.2.5 Analysis of other endpoints 15
5. Flow diagram of trial design 15
6. Adverse events 16
6.1 Definition 16
6.2. Records of adverse events 17
7. Publication of results 17
8. Trial organization 17
8.1 Sponsor 17
8.2 Trial Steering Committee (TSC) 17
8.3 Data Monitoring and Ethics Committee (DMEC) 18

1.Background

Coronary artery bypass grafting (CABG) is a widely performed surgical procedure of significant importance. Despite advancements in surgical techniques, neurocognitive dysfunction remains a major concern for patients recovering from CABG. [1,2] The mechanisms through which coronary artery bypass graft (CABG) surgery impacts cognitive function include several key factors. First, changes in cerebral blood flow occur during the surgical process under cardiopulmonary bypass, where the heart is maintained in a non-beating state to transplant other vessels to the narrow or blocked areas of the coronary arteries, potentially resulting in decreased cerebral blood flow and subsequent cerebral ischemia. Second, cerebrovascular events may arise from the surgeon's manipulation of the coronary arteries or other vessels, potentially leading to the release of embolic materials and causing cerebral infarction or minor cerebrovascular events, thus affecting cognitive function. Third, ischemia/reperfusion can trigger a systemic inflammatory response, causing the release of various inflammatory mediators, impacting the nervous system and increasing the risk of embolism, ultimately leading to cognitive dysfunction. Additionally, patient age is a significant risk factor, particularly with the increasing aging population, as elderly patients undergoing CABG surgery, who often have comorbidities such as hypertension, high cholesterol, and diabetes, are at markedly higher risk of postoperative delirium, cognitive dysfunction, and other neurological and psychiatric complications. Lastly, the complexity and prolonged duration of the surgery itself may also be associated with the risk of neurological complications, particularly due to the extended periods of anesthesia and cardiac arrest, further increasing the duration of cerebral blood flow changes and the overall risk.[3-6] Postoperative delirium (POD), an acute onset, waxing and waning disturbance of consciousness hallmarked by disorganized thinking and inability to focus, sustain or shift attention that typically occurs 24-72 hours after surgery, and cannot be explained by a pre-existing dementia, occurs in 30% to 50% of CABG patients,[7-9] adversely affecting prognosis, quality of life, caregiver burden, and healthcare costs.[10-12]

Understanding the pathophysiology of POD is limited, hindering the development of targeted interventions. The factors contributing to the onset of postoperative delirium, the complexity of diagnosis, numerous complications, and the difficulty in controlling the risk of adverse drug reactions have posed significant challenges to the development and exploration of effective treatment methods. Therefore, the prevention and treatment of postoperative delirium remains a focal and pressing issue. In 2014, the American Geriatrics Society (AGS) and the American College of Surgeons (ACS) jointly released clinical practice guidelines for the prevention and treatment of postoperative delirium.[13] These guidelines, formulated according to the standards of the National Institutes of Health, suggested that no drugs have shown significant therapeutic or preventive effects on postoperative delirium, and thus, safe and effective pharmacological treatments for delirium have yet to be established. The guidelines emphasized the importance of non-pharmacological prevention strategies, medical staff education, medical assessment of delirium causes, optimization of non-opioid pain management strategies, and the avoidance of high-risk medications. Notably, non-pharmacological interventions have consistently proven to be the most effective primary prevention strategy for delirium in surgical patients during non-intensive care hospitalization. [14,15] Therefore, non-pharmacological clinical trials emerged at the beginning of the 21st century, encompassing promoting physical exercise, optimizing existing medical

conditions, or nutritional interventions to mitigate significant risk factors, such as those related to vascular health. [16,17] The most widely studied intervention for delirium in hospitalized non-surgical patients is the Hospital Elder Life Program (HELP).[17] Based on the Yale Delirium Prevention Program, the Hospital Elder Life Program (HELP) implements a standardized protocol targeting six delirium risk factors: pre-existing cognitive impairment, sleep deprivation, immobility, visual impairment, hearing impairment, and dehydration. It has been demonstrated to reduce delirium by 14.4% in medical inpatients, resulting in an estimated cost savings of more than $1.2 million per year in a 500-bed community teaching hospital. Recently, a modified approach based on HELP, emphasizing early post-operative mobilization, nutrition, and cognitive activities, was employed with a surgical population. Among 179 elderly patients undergoing elective abdominal surgery, none experienced postoperative delirium, whereas the control group of 77 patients had an incidence of postoperative delirium of 16.7%.[15]

A series of risk and protective factors have been reported to alter the risk for cognitive decline, mild cognitive impairment or dementia, conceivably via reserve-related mechanisms. [18-23] Cognitive reserve focuses on the idea that there are individual differences in adaptability (i. e. flexibility, efficiency, capacity, compensation) of functional brain processes that allow some people to cope better than others with age-and disease-related brain change, which means cognitive reserve may help compensate for the effects of pathological changes across individual cognitive functions. Original support for the cognitive reserve concept came from epidemiologic studies, primarily in Alzheimer's disease (AD).[24] Presumably cognitive reserve accumulates most during childhood and young adulthood, but it may also build up in older age,[25] which arguably underscores the importance of intellectual engagement throughout the lifespan. A substantial body of evidence suggests that enriched environments facilitate learning and memory capabilities in both human and animal models. Animals living in environments rich in social and cognitive stimuli demonstrate enhanced learning and memory, reduced cellular responses to stress, augmented neurogenesis in the dentate gyrus, increased brain volume and weight, greater dendritic branching, and a rise in synaptic formation.[26-28] Similarly, in humans, factors such as higher education and other forms of cognitive enrichment appear to buffer against cognitive decline and the onset of dementia in older adults, as well as enhance cognitive flexibility.[29] The underlying mechanisms may be akin to those observed in animal studies.

Several studies have taken on the task of identifying ways to augment cognitive reserve. Interventions aimed at exercising cognitive functions typically involve a set of standardized tasks for guided practice, which are designed to reflect specific cognitive functions such as memory, problem solving and information processing speed (ACTIVE Trial).[30] These tasks may be presented in the form of pen-and-paper exercises or computer-based online programs. The ACTIVE trial represents the inaugural large-scale, multi-center clinical study aimed at investigating the influence of cognitive training on cognitive function and daily life capabilities of community elderly. The subsequent 10-year follow-up of the trial conclusively revealed that older adults living in the community who undertook 10 to 14 weeks of structured cognitive training exhibited notable enhancements in cognitive function and demonstrated superior maintenance of daily living activities compared to their untrained counterparts. Although these improvements gradually diminish over time, the effects of memory training endure for a minimum of 5 years, while reasoning and processing speed training maintain impact for at least 10 years. A systematic review and meta-analysis incorporating 25 randomized controlled studies indicated that cognitive exercise training may also enhance the cognitive function of patients with mild cognitive

impairment.[31] Gates et al. demonstrated that cognitive training may attenuate the cognitive decline process in high-risk individuals.[32] Moreover, another meta-analysis suggests that cognitive training leads to measurable improvements in cognitive performance in individuals with Parkinson disease (PD), particularly in working memory, executive functioning, and processing speed, which are typically impaired in the disease. [33] A recent article published in Nature indicated that video games can have lasting benefits on cognitive function after as little as 10 hours of exposure over 2 weeks.[34]

Just recently, cognitive reserve is regarded as a potentially modifiable protective factor that may guard against the development of postoperative delirium and postoperative cognitive dysfunction.[35] Despite strong evidence favoring the ability of cognitive activity (structured and unstructured) to enhance cognitive reserve, our preliminary systematic review and meta-analysis revealed a notable scarcity of studies applying cognitive activity to perioperative patients to counteract postoperative cognitive impairment, particularly in the context of cardiac surgery with postoperative delirium as the primary outcome.[36]Our meta-analysis included only a few randomized controlled trials on perioperative cognitive training, most of which were small-sample feasibility studies. The Prevention of Early Postoperative Decline trial,[37] a feasibility study of 40 patients having cardiac surgery, suggested that patients scheduled for elective cardiac surgery were more likely comply with cognitive training in the preoperative stage, compared to other stages of the perioperative period. Another in-hospital cognitive training trail with consistent supervision, along with allotted breaks to prevent stress and help consolidate learning, provided some meaningful gains in cognitive function.[38] However, this study is also merely a pilot investigation that conducted postoperative cognitive training on a cohort of 50 patients. And finally, a recent randomized trial reported that preoperative cognitive training reduced delirium by 42% in older patients recovering from major noncardiac surgery, which, however, was derived from a post hoc analysis that removed 4 patients who did not meet the minimum adherence criteria.[39] Hence, there is currently still a lack of strong evidence to demonstrate whether cognitive training can be applied to surgical patients and whether it truly can, by enhancing the preoperative cognitive reserve of patients, to some extent, counteract the adverse cognitive effects of surgery and anesthesia, ultimately reducing the risk of postoperative cognitive impairment.

2.Objective

The purpose of this multicenter trial is to study the effectiveness of preoperative cognitive training in preventing postoperative delirium among patients with coronary artery bypass grafting.

2.1Primary Aim

To evaluated the extent to which preoperative in-hospital cognitive training reduces delirium in patients recovering from coronary artery bypass grafting surgery.

2.2 Secondary Aims

(1) To evaluate the feasibility of implementing cognitive training in the preoperative period and the compliance of patients.

(2) To evaluate the effectiveness of preoperative cognitive training in reducing postoperative cognitive dysfunction among patients undergoing coronary artery bypass grafting.
(3) To evaluate whether cognitive training has varying degrees of effect across patients with different baseline cognitive levels.
(4) To evaluate the potential dose-response relationship between the total hours of cognitive training and risk of delirium.
(5) To evaluate the effect of preoperative cognitive training on days alive out of hospital 30 (DAOH30) after surgery, 30-day all-cause mortality compared with routine care.

3. Methods and Study design

3.1 Investigational Plan

Potential research participants are primarily identified through two methods. First, when a patient is diagnosed by the treating surgeon and a specific surgical date is scheduled, the treating physician briefly introduces the research protocol and its objectives to the patient, and inquire about their willingness to participate in the study. If the patient expresses interest and wishes to learn more, the attending physician promptly notifies our research team. Second, as most surgical patients are transferred from the cardiology ward to the cardiac surgery department, our research personnel screen potential candidates for coronary artery bypass graft surgery through the cardiology electronic medical record system. The attending physician notifies our research team upon confirmation of the transfer. Subsequently, the research personnel conduct in-person screening visits with the patients. During the initial screening visits, they comprehensively explain the study's purpose and relevant details, and seek consent from the patient and their family members. Once the patients are determined to be able to comply with the series of assessments and the use of smartphones involved in the study, their eligibility is evaluated based on the study's inclusion criteria. Upon meeting the criteria, the patients or their immediate family members are asked to sign a written informed consent, and are informed of their right to withdraw from the study at any time.

3.2 Inclusion /Exclusion/Drop criteria

3.2.1 Inclusion criteria

(1) Sign the informed consent；
(2) Patients who plan to undergo isolated coronary artery bypass grafting with general anaesthesia；
(3) Adults aged ≥18 years；
(4) Enrolled participants at least 10 days prior to their surgical procedure.

3.2.2 Exclusion criteria

(1) Patients who had a life expectancy <6 months;
(2) A history of psychiatric or neurological disorders (including depression, severe central nervous system depression, schizophrenia, epilepsy, and Parkinson's or Alzheimer's disease);
(3) Significant impairments such as blindness, severe deafness, or dementia that might hinder cognitive

testing;
(4) Prior use of psychotropic or opioid medications, a previous history of delirium before surgery;
(5) A documented history of alcohol abuse or withdrawal within the past six months;
(6) Inability to communicate effectively before surgery due to coma or dementia;
(7) Ongoing participation in another clinical trial;
(8) Planned re-operation within seven days of the first.

3.2.3 Drop criteria

(1) Patients who died before the primary outcome assessment；
(2) Those who do not have any monitoring records or whose CRF records are incomplete；
(3) Surgeons changed the operation plan
(4) Patients who quit the research;

3.3 Research device and procedures

3.3.1 Research device

Name	Manufacturer	Details
Android mobile phone	Redmi 9A Xiaomi Technology Co. LTD	Internet-connected, with a 6.5-inch-diaginal screen
Application “The Light of Future”	V 4.13.1, Beijing Entelligence Technology Co., Ltd.	Offers a range of online games

3.3.2 Cognitive training group

Patients randomized to cognitive training were given an internet-connected Android mobile phone with a 6.5-inch-diaginal screen. The phones were loaded with a dynamic cognitive exercise mobile application (“The Light of Future”, v4.13.1, Beijing Entelligence Technology Co., Ltd.) that offers a range of online games designed to engage and challenge cognitive abilities including memory, imagination, reasoning, reaction time, attention, and processing speed.[40] Tasks and their difficulty were initially tailored to patients’ age and educational level with the goal of balancing cognitive stimulation and enjoyment. Thereafter, task difficulty was automatically adjusted based on users’ performance with the goal of keeping the challenge level within a zone that promoting adherence to the training program while preventing excessive psychological burden and emotional distress associated with overly difficult tasks. The software developer has generously provided 200 complimentary redemption codes that grant access to all games in the " The Light of Future " when redeemed. Each patient has been allocated a unique redemption code, which, when matched with the corresponding patient, enables the technical staff of the software developer to capture all background information during the training process.

The initial step involves entering the patient's baseline information (i. e. age, gender and educational level and so on) into the software. Before the formal training begins, the research staff

explain the operational procedures and guidelines of the software to the patients. Additionally, we select an example game from each cognitive domain to guide the patients on the effective usage of the software. After receiving the instructions, patients are required to independently play six games, each representing a cognitive domain, to assess their proficiency and completion. Formal cognitive training begins only after patients have achieved a basic proficiency level in using the software games. After the first day of training, the research staff meticulously assess the patients' performance to identify any deficiencies or incorrect actions. If found, the research staff promptly inform and rectify them.

Patients were instructed to spend 10 hours on cognitive training. We asked patients to spend at least a full hour per day, over two or three sessions, and that daily sessions include at least one game from each of the six available cognitive domains, more as time permits. A minimum of 10 days of training prior to surgery is needed, equaling a preoperative exercise dose of at least 10 hours. The patients were systematically guided to complete cognitive games in each domain successively. After finishing a game in one cognitive domain, they immediately moved to the next one, and this sequential training ensured that each patient underwent comprehensive exercises in 6 cognitive domains. Participants were free to choose the timing of each training session. The Light of Future software will ensure that each patient is exposed to the same set of games.

Considering that each game includes a tutorial of several tens of seconds, and patients may potentially linger in a game due to various reasons without actively participating but still being automatically timed. Hence, it is necessary to distinguish between the actual in-game time and time spent outside of the game. Because each game is limited by a countdown timer, with each game lasting approximately 58 seconds. We consider the time spent lingering in a game for over two minutes with a completion rate of less than 50%, and those abruptly quitting before reaching 50% completion as invalid training time. If a patient exits a game midway after exceeding 50% completion, the actual effective training duration is calculated based on the completion percentage generated within that game. Additionally, when calculating the actual in-game participation time, we exclude the tutorial time at the beginning of each game.

Details of each participant’s use was recorded by the Light of Future application, including which games were used, when and for how long, and participants’ success with each. At the end of each day's training, the researchers collect the devices and calculate the actual effective training duration for the patients based on detailed backend information. The total training duration is computed by summing up all the training durations the day before the surgery. The final record of the total training duration is in hours (recorded as an integer). For instance, if the total duration is more than 6 hours but less than 7 hours, it is recorded as 6 hours. The evening prior to the surgical procedure, the researchers will comprehensively collect the equipment and complete the collection of all training data. The patient will be unable to participate in any cognitive training.

3.3.3 Routine care group

Participants assigned to routine care were provided with standard hospital attention without any specific cognitive training.

3.4 Randomization Procedure

Patients were assigned by computer to either cognitive training or routine care in a 1:1 ratio stratified by

study site with random blocking. The computer-generated randomization schema was developed by an unblinded statistician and allocation was concealed from study investigators in sequentially numbered sealed opaque envelopes.

3.5 Blinding

Investigators_who provided preoperative cognitive training opened randomization envelopes immediately after eligibility assessment and formal trial enrolment. Patients were not blinded as they needed to adhere to the protocol. However, surgeons, anaesthesiologists, statisticians, and members of the study team responsible for assessing neurocognitive function and postoperative clinical outcomes were unaware of the patient allocation or treatment.

3.6 Anesthesia protocol

3.6.1 Preoperative management

On the day of surgery, shortly before going to the operating room, patients' cognition was assessed using the Montreal Cognitive Assessment (MoCA). After participants entered the operating room, indwelling needle was used to establish intravenous channels. Then lactate Ringer's solution was injected, and vital signs were monitored, including (1) ECG; (2) Non-invasive blood pressure and invasive blood pressure monitoring; (3) Pulse oxygen saturation (SpO2); (4) Nasopharyngeal temperature; (5) Bispectral index (BIS). The baseline values of all monitoring indicators of the patient are recorded after the patient is stabilized (systolic and diastolic blood pressure and heart rate are the basic values at this time).

3.6.2 Protocol of anesthesia

All participants were given routine general anaesthesia induced with etomidate and maintained with sevoflurane in oxygen/air, propofol, fentanyl, and muscle relaxants. BIS was used to monitor the depth of anesthesia, and maintained at 45-60. Neuromuscular block was provided with continuous intravenous infusions or intermittent boluses of cisatracurium. Benzodiazepines, ketamine, and dexmedetomidine were avoided to the extent practical, with other aspects of anaesthetic management left to clinicians' discretion. Blinded investigators reviewed all electronic anaesthesia records to confirm protocol compliance and collect intraoperative data.

3.6.3 Airway management

After patient's eyelash reflex disappeared, muscle relaxation was complete and BIS was stable at 45-60, endotracheal intubation was completed. The volume control ventilation mode is adopted during surgery. The tidal volume was set to 6-8 ml/kg, and respiratory frequency was set to 10-12 times/min. PETCO2 was maintained at 35~45 mmHg.

3.7. Outcome measures

3.7.1 Preoperative measures

(1) Demographic information: age, sex, height, weight, body mass index, education level, cognition;

(2) Medical history: past history, history of anesthesia, ASA classification, comorbidities, diagnoses;
(3) Procedure: type of procedure;
(4) Circulatory indicators: pre-anesthesia vital signs (heart rate, [HR], systolic BP [SBP], diastolic BP [DBP], SpO2, respiratory rate [RR]);
(5) Laboratory examination: Blood routine examination, liver and kidney function;
(6) Mental state: used by Geriatric depression scale;
(7) Preoperative cognitive function status: used by Montreal Cognitive Assessment (MoCA); [41]
(8) Presence of delirium before surgery: used by Confusion Assessment Method (CAM);[42]
(9) Preoperative frailty: used by the Frail Scale.

3.7.2 Intraoperative measures

(1) The use of anesthetic drugs and vasoactive agents;
(2) intraoperative fluid balance;
(3) Dosage of Midazolam;
(4) Dosage of Dexmedetomidine;
(5) Surgical procedures (with cardiopulmonary bypass or without);
(6) No. of distal anastomosis;
(7) Length of surgery;
(8) Length of anesthesia;
(9) Hemodynamic information: (heart rate, [HR], mean arterial pressure (MAP), central venous pressure (CVP) systolic BP [SBP], diastolic BP [DBP], SpO_2, respiratory rate [RR], BIS value, $ETCO_2$).

All intraoperative data were obtained by review of medical records and anesthesia records.

3.7.3 Postoperative outcomes

The primary outcome:

The incidence of delirium from postoperative day 1 to postoperative day 7 or before discharge, whichever occurred first.

Diagnosis of POD was based on the results of CAM or CAM-ICU twice daily, [42,43] specifically from 08:00 to 10:00 and from 18:00 to 20:00. All blinded researchers were trained in CAM/CAM-ICU assessment and the consistency among research members was reached. When there is a contradiction between the diagnosis obtained by the scale and the diagnosis of the psychiatrist, when there are still differences after consultation, the diagnosis of the psychiatrist shall prevail. In order to prevent missed diagnosis, we should communicate with the medical staff in the ward in time. After the patient is selected, the family member or escort staff should be informed, and the investigator should be informed of the evaluation of delirium in time when any abnormal performance of the patient's mood, consciousness, cognition and so on is found. Comprehensive reviews of progress notes, nursing documents, and medical records were conducted by blinded investigators to identify instances of delirium between postoperative day 1 and 7, employing a chart-based delirium identification instrument. [44,45] Patients diagnosed with delirium through any of the aforementioned methods were considered to have experienced delirium on that particular day. When postoperative delirium is found, the subject should communicate with the competent physician and decide on further diagnosis and treatment. If the patient's performance was normal 7 d after operation, the visit ended, while the patient with continuous delirium was followed up

until normal or discharged.
Outcomes were assessed by investigators specifically trained in recognition and assessment of delirium.

Secondary outcomes:
(1) cumulative incidence of POD
(2) incidence of severe delirium, evaluated with the Memorial Delirium Assessment Scale (MDAS)[46]
(3) postoperative cognitive dysfunction (POCD) on postoperative day 7 or at discharge if earlier, used by the Montreal Cognitive Assessment (MoCA) (range, 0 [worst] to 30 [best]), defined as a 1 standard deviation decrease from baseline in Montreal cognitive assessment score at postoperative day 7 or discharge.
(4) cognitive function one month after surgery, used by the Revised Telephone Interview for Cognitive Status (TICS-m) (range, 0 [worst] to 50 [best])
(5) 30-day all-cause mortality
(6) durations of intensive care unit (number of days in the intensive care unit from the end of operation to discharge) and postoperative hospital care.
(7) elapsed time from the end of surgery to delirium onset
(8) duration of delirium (defined as the period between the first and last delirium-positive day, even if there were non-delirious days in between)
(9) the total number of delirium-positive days in patients who developed delirium
(10) cognitive function at three time points (baseline, preoperative, postoperative day 7or at discharge if earlier. was used to assess early POCD on the seventh day after surgery or at discharge and cognitive function at any time points before or after surgery.
(11) Postoperative intubation time, defined as duration from the end of the procedure to extubation.
(12) intensive care unit readmission within 30 days after surgery.
(13) Number of days alive and out of the hospital within 30 days. (The sum of the number of days patients were alive and not in the hospital within 30 days after surgery. It is calculated as 30 days minus the total number of days in hospital after surgery (maximum 30 days, minimum 0 days). If the patient is not hospitalized from 00:00 to 24:00 in a day, the number of days without hospitalization can be counted; After discharge, patients were hospitalized for a second time, and the second hospitalization time was also included in the total number of days in hospital. The patient died within 30 days of surgery. DAOH 30 is 0 days; If the patient was not discharged 30 days after surgery, DAOH 30 was recorded as 0 days.)

All outcomes were assessed by investigators masked to patient allocation.

4. Statistical Analysis Plan

4.1 Sample size calculation

In a previous study of patients recovering from coronary artery bypass graft surgery, the incidence of postoperative delirium was 35%.[47] We powered our trial to detect a 50% relative reduction in delirium from a 35% baseline consequent to cognitive training. 96 patients were required in each group for a two-tailed α=0.05 with 80% power. Assuming a potential dropout rate of 10%, we included a total of 214 patients (107 in each group).

4.2 Statistical methods

4.2.1 Data set

Modified intention-to-treat analysis (m ITT) was defined that patients were primarily analyzed within the groups to which they were assigned, whether or not the minimum compliance threshold of training was met, excluding those without any record of follow-up or cancelled surgeries. Primary indicators are analyzed according to the m ITT principle.

Per protocol set (PPS) refers to the set of cases that meet the minimum compliance threshold of training, have completed the treatment protocol. The PPS is a subset of the ITT in which each subject in the dataset is a valid case or sample with good adherence, no protocol violations, and complete baseline values for key indicators.

4.2.2 Data presentation

Quantitative data: Kolmogorov-Smirnov test was used to estimate the distribution of continuous variables. Data will be presented using mean ± standard deviation or median (upper and lower quartiles, IQR) according to the normality of data.

Categorical data: Data will be presented using frequencies, composition ratios, or percentages.

4.2.3 General considerations

Data set: The assessment of primary endpoint will be performed based on m ITT set. The per protocol set is mainly used for sensitivity analysis. Patients who engaged in cognitive training for 3 or more hours were considered to have met our minimum compliance threshold, and included in per protocol set. The establishment of this threshold is derived from the results of our preliminary meta-analysis.

Covariates and subgroup analysis: Subgroup analysis will be performed and the subgroup analysis will be carried out as exploratory. To evaluate treatment effect heterogeneity on the primary outcome, predefined subgroup analyses were performed based on surgical methods, age, sex, education level, and baseline cognitive function. Interactions between cognitive training and each variable were assessed by adding interaction terms to the logistic regression model.

multi-center effect analysis: This is a multicenter RCT study, and there may be center effects among different centers. The logistic regression models will be used to control the multi-center effect, with the different centers set as fixed effect.

General analysis:
The data analyses mainly include statistical description and statistical inference. Quantitative data will be described by central tendency and dispersion tendency. The normally distributed data of central tendency and dispersion tendency will be described as means and standard division, respectively. The non-normally distributed data of central tendency and dispersion tendency will be described as median and quartiles. The qualitative data will be described as frequency and percentage. Statistical inference,

independent t test or non-parameter test will be used to compare the quantitative data between the two groups, while chi-square test or Fisher's exact test will be used for comparing the qualitative data between the two groups.

For missing data, imputation techniques were used. Statistical analyses were performed using SPSS 23.0 (IBM SPSS) and R (R software 4.2.1), and 2-sided $P<0.05$ was considered to be statistically significant in all statistical tests.

4.2.4 Analysis of primary endpoint

The incidence of POD between the two groups was compared with χ^2 tests, and the odds ratio (OR)with 95% CI was calculated.

The time-to-event analytic approach was also used to compared the cumulative incidence of POD between the groups. Kaplan-Meier analysis was used to estimate the cumulative incidence of POD, and difference of incidence between groups was compared with the log-rank test.

4.2.5 Analysis of other endpoints

For secondary and exploratory endpoints, continuous data will be presented as means (SDs) or median (IQRs), as appropriate. The secondary endpoint followed no Gaussian distribution, will be presented as median (interquartile range) and tested by Mann-Whitney U test. POCD was defined as a 1 standard deviation decline in MoCA score on postoperative day 7, or at discharge if earlier, compared to baseline. This definition aligns with previous definitions of POCD and current recommendations for defining POCD or delayed neurocognitive recovery. [48,49] Mantel-Haenszel χ^2 and Cuzick tests assessed a potential dose-response relationship between the total hours of cognitive training and incidence of delirium. Analyses of delirium duration and delirium-positive days were conducted using the Zero-inflated Poisson regression model. Regarding the assessment of delirium severity, ordinal logistic regression model was used to model no delirium, delirium and severe delirium. Additionally, a post-hoc analysis incorporating study site, age, surgical technique, baseline educational level and cognitive function of the participants was conducted.

5. Flow diagram of trial design

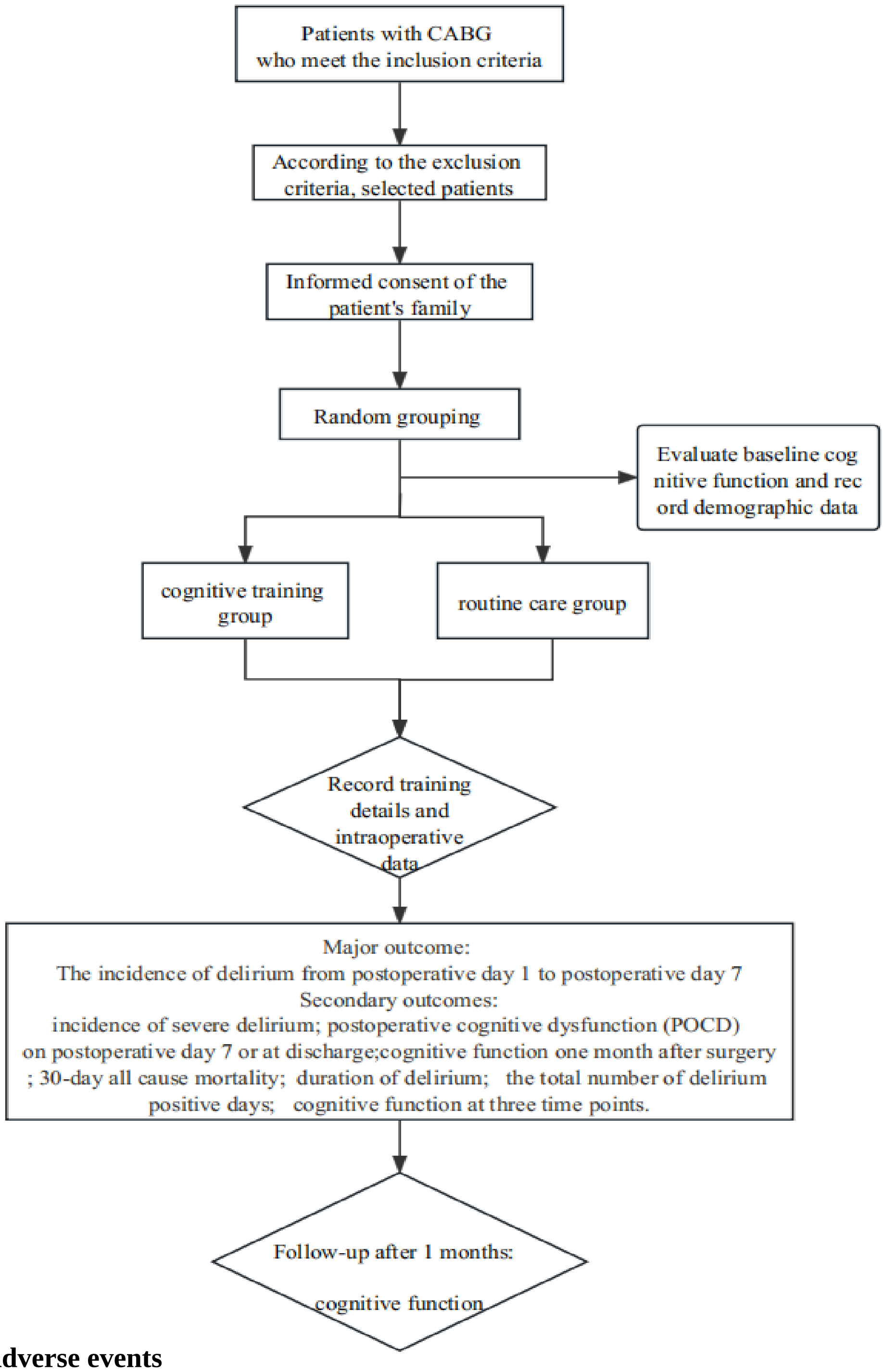

6. Adverse events

6.1 Definition

Adverse events (Adverse Event, AE) refer to any adverse symptoms, abnormal signs and abnormal

laboratory results after the intervention study, whether or not there is a causal relationship with the intervention. Any adverse events, including those provided by the subjects voluntarily or obtained through inquiry by the investigator or by physical examination, laboratory examination or other examination methods, should be recorded on the CRF and actively handled, followed closely until remission or condition is stable.

6.2. Records of adverse events

(1) All adverse events must be tracked, and the investigator must record them in the corresponding section of the case report form and must record the adverse events in detail.
(2) Adverse event content: name of symptom or sign or diagnostic name of disease or laboratory examination of an indicator abnormality.
(3) Date of onset of adverse events: date of first occurrence of AE or related symptoms AE subjects.
(4) End date of adverse events: date of cessation of AE or AE-related symptoms. If the AE still exists, do not fill in the end date.
(5) Evaluation of the severity of adverse events a. Mild: symptoms and signs present, but can tolerate, generally without treatment; b. Moderate: symptoms or signs cause discomfort, daily activities are limited, appropriate treatment can be taken; c. Severe: no ability to work and daily activities, need active treatment.
(6) Measures taken: a. discontinuation of the relevant medication or treatment; b. use of therapeutic measures; c. no measures taken.
(7) End result of adverse events: persistence, improvement, mitigation.
(8) Evaluation of the correlation between adverse events and intervention.

7. Publication of results

All research participants shall strictly keep confidential all information provided by The First Affiliated Hospital of Anhui Medical University, and at the same time require other participants and ethics committees to take the same confidentiality measures, without written permission, Information shall not be leaked to others. All the data and results of this experiment are owned by the investigators and the sponsor. The investigators may not publish himself/herself without the consent of the applicant. The sponsor reserves the right to sign the paper. The sponsor is the unit to which the project results belong.

8. Trial organization

8.1 Sponsor

The leader sponsor: The First Affiliated Hospital of Anhui Medical University, Hefei, Anhui, China.

8.2 Trial Steering Committee (TSC)

Face to face meeting will be held at regular intervals determined by need. Routine business is conducted

by email, offline meeting or teleconferencing. The Steering Committee, in the development of this protocol and throughout the trial will take the responsibility for: a. Major decisions such as a need to change the protocol for any reason; b. Monitoring and supervising the progress of the trail; c. Reviewing relevant information from other sources; d. Considering recommendations from DMEC; e. Informing and advising on all aspects of the trial.

8.3 Data Monitoring and Ethics Committee (DMEC)

The DMEC will advise the Chairman of the Steering Committee if, in their view, the randomized comparisons have provided both (1) 'proof beyond reasonable doubt' that for all, or some, the treatment is clearly indicated or clearly contra-indicated and (2) evidence that might reasonably be expected to materially influence future patient management. Following a report from the DMEC, the Steering Committee will decide what actions, if any, are required. Unless the DMEC request cessation of the trial the Steering Committee and the collaborators will remain ignorant of the interim results.

References

1.Tachibana H, Hiraoka A, Saito K, et al. Incidence and impact of silent brain lesions after coronary artery bypass grafting. The Journal of thoracic and cardiovascular surgery. 2021;161(2):636-644.

2.Greaves D, Psaltis PJ, Ross TJ, et al. Cognitive outcomes following coronary artery bypass grafting: A systematic review and meta-analysis of 91,829 patients. International journal of cardiology. 2019; 289: 43-49.

3.Kok WF, Koerts J, Tucha O, Scheeren TW, Absalom AR. Neuronal damage biomarkers in the identification of patients at risk of long-term postoperative cognitive dysfunction after cardiac surgery. Anaesthesia. 2017;72(3):359-369.

4.Roach GW, Kanchuger M, Mangano CM, et al. Adverse cerebral outcomes after coronary bypass surgery. Multicenter Study of Perioperative Ischemia Research Group and the Ischemia Research and Education Foundation Investigators. *The New England journal of medicine.* 1996;335(25):1857-1863.

5. Bhushan S, Li Y, Huang X, Cheng H, Gao K, Xiao Z. Progress of research in postoperative cognitive dysfunction in cardiac surgery patients: A review article. *International journal of surgery (London, England).* 202; 95:106163.

6.Guerrieri Wolf L, Abu-Omar Y, Choudhary BP, Pigott D, Taggart DP. Gaseous and solid cerebral microembolization during proximal aortic anastomoses in off-pump coronary surgery: the effect of an aortic side-biting clamp and two clampless devices. *The Journal of thoracic and cardiovascular surgery.* 2007;133(2):485-493.

7.Kazmierski J, Banys A, Latek J, Bourke J, Jaszewski R. Raised IL-2 and TNF-alpha concentrations are associated with postoperative delirium in patients undergoing coronary-artery bypass graft surgery. Int Psychogeriatr 2013;1–11.

8.Mu DL, Wang DX, Li LH, Shan GJ, Li J, Yu QJ, et al. High serum cortisol level is associated with increased risk of delirium after coronary artery bypass graft surgery: a prospective cohort study. Crit Care 2010;14: R238-248.

9.Szwed K, Pawliszak W, Szwed M, Tomaszewska M, Anisimowicz L, Borkowska A. Reducing delirium and cognitive dysfunction after off-pump coronary bypass: A randomized trial. J Thorac Cardiovasc Surg. 2021;161(4):1275-1282.e4. doi:10.1016/j. jtcvs.2019.09.081.

10.Salluh JI, Wang H, Schneider EB, et al. Outcome of delirium in critically ill patients: systematic review and meta-analysis. *BMJ (Clinical research ed).* 2015;350:h2538.

11.Huang H, Li H, Zhang X, et al. Association of postoperative delirium with cognitive outcomes: A meta-analysis. *Journal of clinical anesthesia.* 2021; 75:110496.

12.Sauër AC, Veldhuijzen DS, Ottens TH, Slooter AJC, Kalkman CJ, van Dijk D. Association between delirium and cognitive change after cardiac surgery. *British journal of anaesthesia.* 2017;119(2):308-315.

13.American Geriatrics Society abstracted clinical practice guideline for postoperative delirium in older adults. *Journal of the American Geriatrics Society* 2015; **63**(1): 142-50.

14. Chen CC, Saczynski J, Inouye SK. The modified Hospital Elder Life Program: adapting a complex intervention for feasibility and scalability in a surgical setting. *J Gerontol Nurs* 2014; **40**(5): 16-22.

15. Chen CC, Li HC, Liang JT, et al. Effect of a Modified Hospital Elder Life Program on Delirium and Length of Hospital Stay in Patients Undergoing Abdominal Surgery: A Cluster Randomized Clinical Trial. *JAMA surgery* 2017; **152**(9): 827-34.

16.Deeken F, Sánchez A, Rapp MA, et al. Outcomes of a Delirium Prevention Program in Older Persons After Elective Surgery: A Stepped-Wedge Cluster Randomized Clinical Trial. *JAMA surgery.* 2022;157(2): e216370.

17. Rubin FH, Williams JT, Lescisin DA, Mook WJ, Hassan S, Inouye SK. Replicating the Hospital Elder Life Program in a community hospital and demonstrating effectiveness using quality improvement methodology. *J Am Geriatr Soc.* 2006;54(6):969-74.

18.Chapko D, McCormack R, Black C, Staff R, Murray A. Life-course determinants of cognitive reserve (CR) in cognitive aging and dementia–a systematic literature review. Aging Ment Health. 2018;22(8):915–26.

19. Meng X, D'Arcy C. Education and dementia in the context of the cognitive reserve hypothesis: a systematic review with meta-analyses and qualitative analyses. PLoS One. 2012;7(6): e38268.

20. Valenzuela MJ, Sachdev P. Brain reserve and dementia: a systematic review. Psychological Med. 2006;36(4):441–54.

21. Scarmeas N, Stern Y. Cognitive reserve and lifestyle. J Clin Exper Neuropsychol. 2003;25(5):625–33.

22. Christie GJ, Hamilton T, Manor BD, Farb NAS, Farzan F, Sixsmith A, Temprado JJ, Moreno S. Do lifestyle activities protect against cognitive decline in aging? A review. Front Aging Neurosci. 2017; 9:381.

23. Harrison SL, Sajjad A, Bramer WM, Ikram MA, Tiemeier H, Stephan BC. Exploring strategies to operationalize cognitive reserve: a systematic review of reviews. J Clin Exp Neuropsychol. 2015;37(3):253–64.

24. Stern Y, Gurland B, Tatemichi TK, Tang MX, Wilder D, Mayeux R. Influence of education and occupation on the incidence of Alzheimer's disease. JAMA. 1994;271(13):1004–10.

25.Cabeza R, Albert M, Belleville S, et al. Maintenance, reserve and compensation: the cognitive neuroscience of healthy ageing. *Nature reviews Neuroscience.* 2018;19(11):701-710.

26. Leggio MG, Mandolesi L, Federico F, et al. Environmental enrichment promotes improved spatial abilities and enhanced dendritic growth in the rat. *Behavioural brain research.* 2005;163(1):78-90.

27. Nithianantharajah J, Hannan AJ. Enriched environments, experience-dependent plasticity and disorders of the nervous system. Nat Rev Neurosci. 2006;7(9):697–709. 120.

28. Sale A, Berardi N, Maffei L. Enrich the environment to empower the brain. Trends Neurosci. 2009;32(4):233–9.

29.Stern Y. Cognitive reserve in ageing and Alzheimer's disease. *The Lancet Neurology.* 2012;11(11):1006-1012.

30. Ball K, Berch DB, Helmers KF, Jobe JB, Leveck MD, Marsiske M, Morris JN, Rebok GW, Smith DM, 1221 Tennstedt SL, Unverzagt FW, Willis SL; Advanced Cognitive Training for Independent and Vital Elderly Study Group. Effects of cognitive training interventions with older adults: a randomized controlled trial. JAMA. 2002;288(18):2271-81.

31. Hill N T M, Mowszowski L, Naismith S L, et al. Computerized cognitive training in older adults with mild cognitive impairment or dementia: a systematic review and meta-analysis. American Journal of Psychiatry. 2017; 174(4): 329-340.

32. Gates NJ, Sachdev PS, Fiatarone Singh MA, Valenzuela M. Cognitive and memory training in adults at risk of dementia: a systematic review. *BMC geriatrics*. 2011; 11: 55.

33. Leung IH, Walton CC, Hallock H, Lewis SJ, Valenzuela M, Lampit A. Cognitive training in Parkinson disease: A systematic review and meta-analysis. *Neurology*. 2015;85(21):1843-1851.

34. Bavelier D and Davidson RJ. Brain training: Games to do you good. Nature. 2013; 494(7438):425-6.

35. Berger M, Terrando N, Smith SK, Browndyke JN, Newman MF, Mathew JP. Neurocognitive Function after Cardiac Surgery: From Phenotypes to Mechanisms. *Anesthesiology.* 2018;129(4):829-851.

36. Jiang Y, Fang P, Shang Z, Zhu W, Gao S, Liu X. et al. Cognitive training in surgical patients: a systematic review and meta-analysis. Anesthesiol Perioper Sci. 2023;1(3):18.

37. O'Gara BP, Mueller A, Gasangwa DVI, et al. Prevention of Early Postoperative Decline: A Randomized, Controlled Feasibility Trial of Perioperative Cognitive Training. *Anesthesia and analgesia.* 2020;130(3):586-595.

38. Cheng CM, Chiu MJ, Wang JH, et al. Cognitive stimulation during hospitalization improves global cognition of older Taiwanese undergoing elective total knee and hip replacement surgery. *Journal of advanced nursing.* 2012;68(6):1322-1329.

39. Humeidan ML, Reyes JC, Mavarez-Martinez A, et al. Effect of Cognitive Prehabilitation on the Incidence of Postoperative Delirium Among Older Adults Undergoing Major Noncardiac Surgery: The Neurobics Randomized Clinical Trial. *JAMA surgery.* 2021;156(2):148-156.

40. Huang L. A quasi-comprehensive exploration of the mechanisms of spatial working memory. *Nature*

human behaviour. 2023;7(5):729-739.

41. Nasreddine ZS, Phillips NA, Bédirian V, et al. The Montreal Cognitive Assessment, MoCA: a brief screening tool for mild cognitive impairment. *Journal of the American Geriatrics Society.* 2005;53(4):695-699.

42. Inouye SK, van Dyck CH, Alessi CA, Balkin S, Siegal AP, Horwitz RI. Clarifying confusion: the confusion assessment method. A new method for detection of delirium. *Annals of internal medicine.* 1990;113(12):941-948.

43. Chen TJ, Chung YW, Chang HR, et al. Diagnostic accuracy of the CAM-ICU and ICDSC in detecting intensive care unit delirium: A bivariate meta-analysis. *International journal of nursing studies.* 2021; 113:103782.

44. Inouye SK, Leo-Summers L, Zhang Y, Bogardus ST, Jr., Leslie DL, Agostini JV. A chart-based method for identification of delirium: validation compared with interviewer ratings using the confusion assessment method. *Journal of the American Geriatrics Society.* 2005;53(2):312-318.

45. Saczynski JS, Kosar CM, Xu G, et al. A tale of two methods: chart and interview methods for identifying delirium. *Journal of the American Geriatrics Society.* 2014;62(3):518-524.

46. Breitbart W, Rosenfeld B, Roth A, Smith MJ, Cohen K, Passik S. The Memorial Delirium Assessment Scale. *Journal of pain and symptom management.* 1997;13(3):128-137.

47. Szwed K, Pawliszak W, Szwed M, Tomaszewska M, Anisimowicz L, Borkowska A. Reducing delirium and cognitive dysfunction after off-pump coronary bypass: A randomized trial. *The Journal of thoracic and cardiovascular surgery.* 2021;161(4):1275-1282.e1274.

48. Evered L, Silbert B, Knopman DS, et al. Recommendations for the nomenclature of cognitive change associated with anaesthesia and surgery-2018. *British journal of anaesthesia.* 2018;121(5):1005-1012.

49. Berger M, Nadler JW, Browndyke J, et al. Postoperative Cognitive Dysfunction: Minding the Gaps in Our Knowledge of a Common Postoperative Complication in the Elderly. *Anesthesiology clinics.* 2015;33(3):517-550.
