# Supplementary material for: Cognitive Training for Reduction of Delirium in Patients Undergoing Cardiac Surgery: A Randomized Clinical Trial
Source: JAMA Netw Open. 2024 Apr 23;7(4):e247361. doi: 10.1001/jamanetworkopen.2024.7361 (PMC11040409; doi:10.1001/jamanetworkopen.2024.7361)
Supplement: Supplement 2. — eTable 1. Recruitment by Centre eTable 2. Intraoperative Measures and Surgical Characteristics eTable 3. Baseline Characteristics of Patients by Cognitive Training Time eFigure 1. The Light of Future Gameplay Distribution. eTable 4. Post-Hoc Analysis Incorporating Baseline Variables eTable 5. Per-Protocol Analysis of Primary Outcomes eFigure 2. Cumulative Incidence of Delirium in Patients Randomized to Routine Care (Blue) and Cognitive Training (Red) eFigure 3. Postoperative Delirium Onset and Duration eFigure 4. MoCA Scores Over Time eTable 6. Potential Association Between the Total Hours of Cognitive Training and the Risk of Delirium eFigure 5. Association of the Duration of Cognitive Training and the Risk of Delirium [file jamanetwopen-e247361-s002.pdf]

Supplemental Online Content

Jiang Y, Xie Y, Fang P, et al; CT-LIFE Study Collaborators. Cognitive training for reduction of delirium in patients undergoing cardiac surgery: a randomized clinical trial. *JAMA Netw Open.* 2024;7(4):e247361. doi:10.1001/jamanetworkopen.2024.7361

eTable 1. Recruitment by Centre

eTable 2. Intraoperative Measures and Surgical Characteristics

eTable 3. Baseline Characteristics of Patients by Cognitive Training Time

eFigure 1 . The Light of Future Gameplay Distribution.

eTable 4. Post-Hoc Analysis Incorporating Baseline Variables

eTable 5. Per-Protocol Analysis of Primary Outcomes

eFigure 2. Cumulative Incidence of Delirium in Patients Randomized to Routine Care (Blue) and Cognitive Training (Red)

eFigure 3. Postoperative Delirium Onset and Duration

eFigure 4. MoCA Scores Over Time

eTable 6. Potential Association Between the Total Hours of Cognitive Training and the Risk of Delirium

eFigure 5. Association of the Duration of Cognitive Training and the Risk of Delirium

This supplemental material has been provided by the authors to give readers additional information about their work.

eTable 1. Recruitment by Centre

Center	Full Analysis Set Total = 208 N (% of total)	Per protocol Set Total = 202 N (% of total)	Cognitive training (n = 102)	Routine care (n = 106)
The First Affiliated Hospital of Anhui Medical University	51 (24.5%)	48 (23.8%)	22	29
The First Affiliated Hospital of University of Science and Technology of China	137 (65.9%)	134 (66.3%)	69	68
Nanjing First Hospital Affiliated to Nanjing Medical University	20 (9.6%)	20 (9.9%)	11	9

© 2024 Jiang Y et al. *JAMA Network Open.*

eTable 2. Intraoperative Measures and Surgical Characteristics

Characteristic	Cognitive training (n = 102)	Routine care (n = 106)	*P* value
Intraoperative measures [a]			
Duration of BIS < 40, median [IQR], min	15 (10-25)	20 (15-30)	0.33
No. (%)	35 (34.3)	37 (34.9)	0.93
Duration of MAP < 75, median [IQR], min	20 (15-30)	20 (15-35)	0.75
No. (%)	79 (77.5)	78 (73.6)	0.52
Duration of ETCO2 < 32, median [IQR], min	15 (10-20)	15 (10-20)	0.75
No. (%)	70 (68.6)	72 (67.9)	0.91
Intraoperative medications			
Vasopressors, No. (%)	55 (53.9)	60 (56.6)	0.70
vasodilators, No. (%)	17 (16.7)	20 (18.9)	0.68
Midazolam, No. (%)	24 (23.5)	17 (16.0)	0.18
Dexmedetomidine, N (%)	77 (75.5)	76 (71.7)	0.54
Surgical procedures			
With CPB, No. (%)	11 (10.8)	10 (9.4)	0.75
Without CPB, No. (%)	91 (89.2)	96 (90.6)	
No. of distal anastomosis, No. (%)			
1	2 (2.0)	2 (1.9)	
2	14 (13.7)	15 (14.1)	
3	42 (41.2)	44 (41.5)	0.83
4	37 (36.3)	40 (37.7)	
5	7 (6.9)	5 (4.7)	
Length of surgery, min	304 (260-370)	301 (254-360)	0.87

© 2024 Jiang Y et al. *JAMA Network Open.*

Characteristic	Cognitive training (n = 102)	Routine care (n = 106)	*P* value
Length of anesthesia, min	362 (320-418)	365 (302-415)	0.60

Abbreviations: No, number of patients; BIS, bispectral index; MAP, mean arterial pressure; $ETCO_2$, end-tidal carbon dioxide; CPB, cardiopulmonary bypass.

[a] Intraoperative measurements restricted to off-pump periods.

© 2024 Jiang Y et al. *JAMA Network Open.*

eTable 3. Baseline Characteristics of Patients by Cognitive Training Time

Characteristic	N (%) Cognitive training (n = 102)	≤ 5 Hours (n = 34)	> 5 Hours (n = 68)	*P* value
Age, median [IQR], y	65 (58-70)	65 (59-70)	65 (58-71)	0.93
ASA physical status level				
I-II	3 (2.9)	2 (5.9)	1 (1.5)	
III	76 (74.5)	27 (79.4)	49 (72.1)	0.16
IV	23 (22.5)	5 (14.7)	18 (26.5)	
Education Level				
Primary school or below	43 (42.2)	15 (44.1)	28 (41.2)	
Middle school	29 (28.4)	9 (26.5)	20 (29.4)	0.94
High school or above	30 (29.4)	10 (29.4)	20 (29.4)	
Frailty score, median [IQR]	1 (0-2)	1 (0-1)	1 (0-2)	0.19
Charlson Comorbidity Index score, median [IQR]	1 (0-2)	1 (0-1)	1 (0-2)	0.19
Montreal Cognitive Assessment, median [IQR]	26 (25-28)	26 (25-28)	27 (25-28)	0.34
Geriatric Depression Scale, median [IQR]	0 (0-1)	0 (0-1)	0 (0-1)	0.71
Mild Cognitive Impairment	39 (38.2)	15 (44.1)	24 (35.3)	0.39

Abbreviations: ASA, American Society of Anesthesiologist classification; IQR, interquartile range

Data are presented as numbers (%), means ± SDs, or medians (interquartile range).

© 2024 Jiang Y et al. *JAMA Network Open.*

eFigure 1. The Light of Future Gameplay Distribution

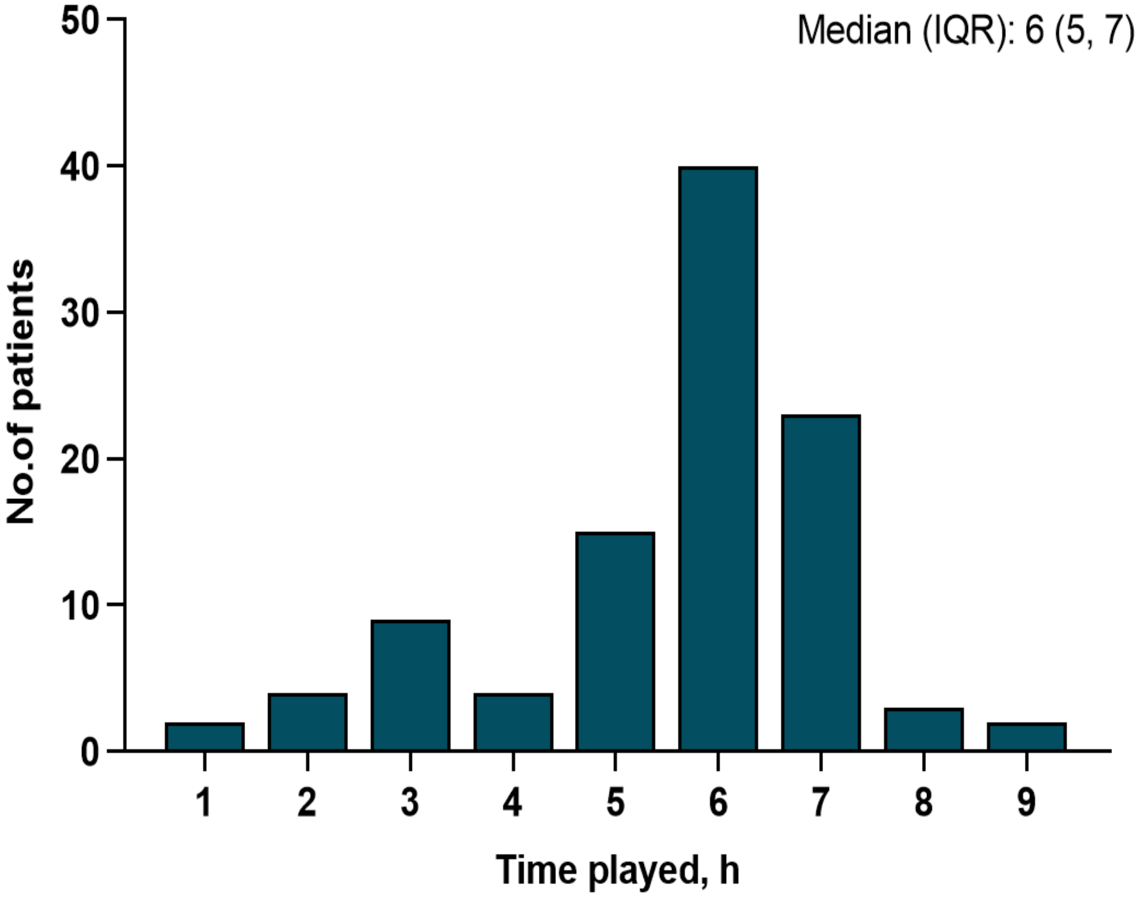

Abbreviations: IQR, interquartile ranges; h, hour; No, number.

The duration of preoperative training ranged from 1 to 9 hours and median and interquartile ranges (IQR) of training time was 6 (IQR, 5-7) hours. Six patients assigned to cognitive training participated in less than 3 hours of cognitive training before surgery.

© 2024 Jiang Y et al. *JAMA Network Open.*

eTable4. Post-Hoc Analysis Incorporating Baseline Variables

Characteristic	No. (%)		Adjusted Odds Ratio (95% CI) [c]	Adjusted *P* value [c]
	Cognitive training (n = 102)	Routine care (n = 106)		
**Primary outcome**				
Postoperative delirium	28 (27.5)	46 (43.4)	0.43 (0.23-0.77)	0.007
**Secondary outcome**				
Delirium onset, postoperative d				
0 to 1	13 (12.7)	22 (20.8)	0.51 (0.23-1.10)	0.09
0 to 2	27 (26.5)	41 (38.7)	0.51 (0.27-0.95)	0.04
0 to 7	28 (27.5)	46 (43.4)	0.43 (0.23-0.77)	0.007
Severe delirium [a]	13 (12.7)	17 (16.0)	0.46 (0.25-0.83)	0.01
Delirium duration, median (IQR), d [b]	0 (0-1)	0 (0-2)	NA	0.008
Delirium-positive days, median (IQR), d [b]	0 (0-1)	0 (0-2)	NA	0.005

Abbreviations: IQR, interquartile range; CI, confidence interval; NA, not applicable.

[a] Ordinal logistic regression model was used to model no delirium, delirium and severe delirium simultaneously on all patients.

[b] Analysis of delirium duration and delirium-positive days was conducted using the Zero-inflated Poisson regression model.

[c] Analysis were adjusted for study site, age, surgical technique, baseline educational level and cognitive function of the participants.

© 2024 Jiang Y et al. *JAMA Network Open.*

eTable 5. Per-Protocol Analysis of Primary Outcome

Characteristic	No. (%)		Odds Ratio (95% CI)		*P* value adjusted for center
	Cognitive training (n = 96)	Routine care (n = 106)	Unadjusted	Adjusted for center	
**Primary outcome**					
Postoperative delirium	25 (26.0)	46 (43.4)	0.46 (0.25-0.83)	0.38 (0.20-0.71)	0.003
**Secondary outcome**					
Delirium onset, postoperative d					
0 to 1	12 (12.5)	22 (20.8)	0.55 (0.25-1.16)	0.47 (0.21-1.03)	0.06
0 to 2	24 (25.0)	41 (38.7)	0.53 (0.29-0.96)	0.46 (0.24-0.85)	0.02
0 to 7	25 (26.0)	46 (43.4)	0.46 (0.25-0.83)	0.38 (0.20-0.71)	0.003
Severe delirium	12 (12.5)	17 (16.0)	0.49 (0.28-0.89)	0.41 (0.22-0.76)	0.005
Delirium duration, median (IQR), d	0 (0-1)	0 (0-2)	NA	NA	0.004
Delirium-positive days, median (IQR), d	0 (0-1)	0 (0-2)	NA	NA	0.003

© 2024 Jiang Y et al. *JAMA Network Open.*

eFigure 2. Cumulative Incidence of Delirium in Patients Randomized to Routine Care (Blue) and Cognitive Training (Red)

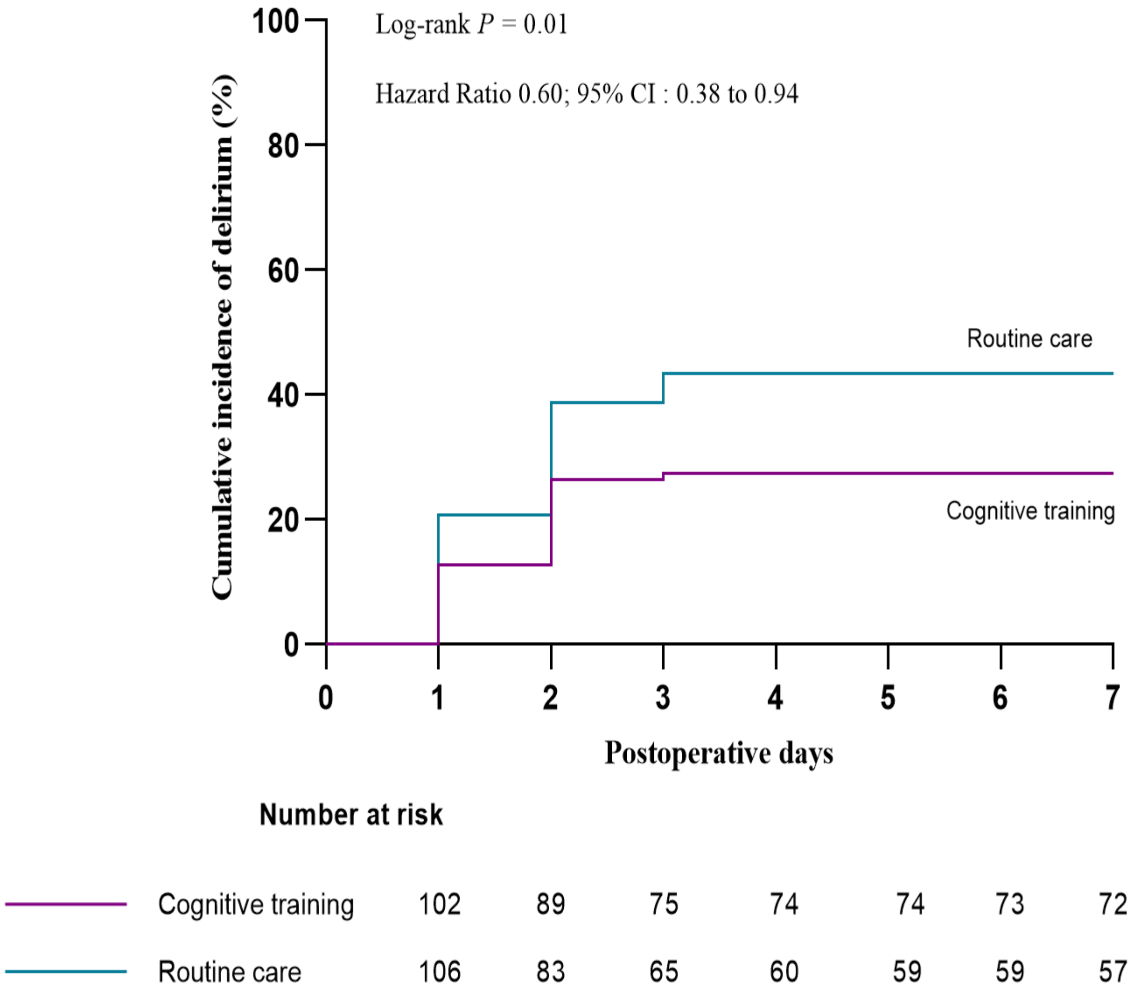

Cumulative delirium incidence over postoperative days 1-7: Log-rank (Mantel-Cox) test: P = 0.01; Hazard ratio = 0.60, 95% CI, 0.38 to 0.94
Abbreviations: CI, confidence interval.

© 2024 Jiang Y et al. *JAMA Network Open.*

eFigure 3. Postoperative Delirium Onset and Duration

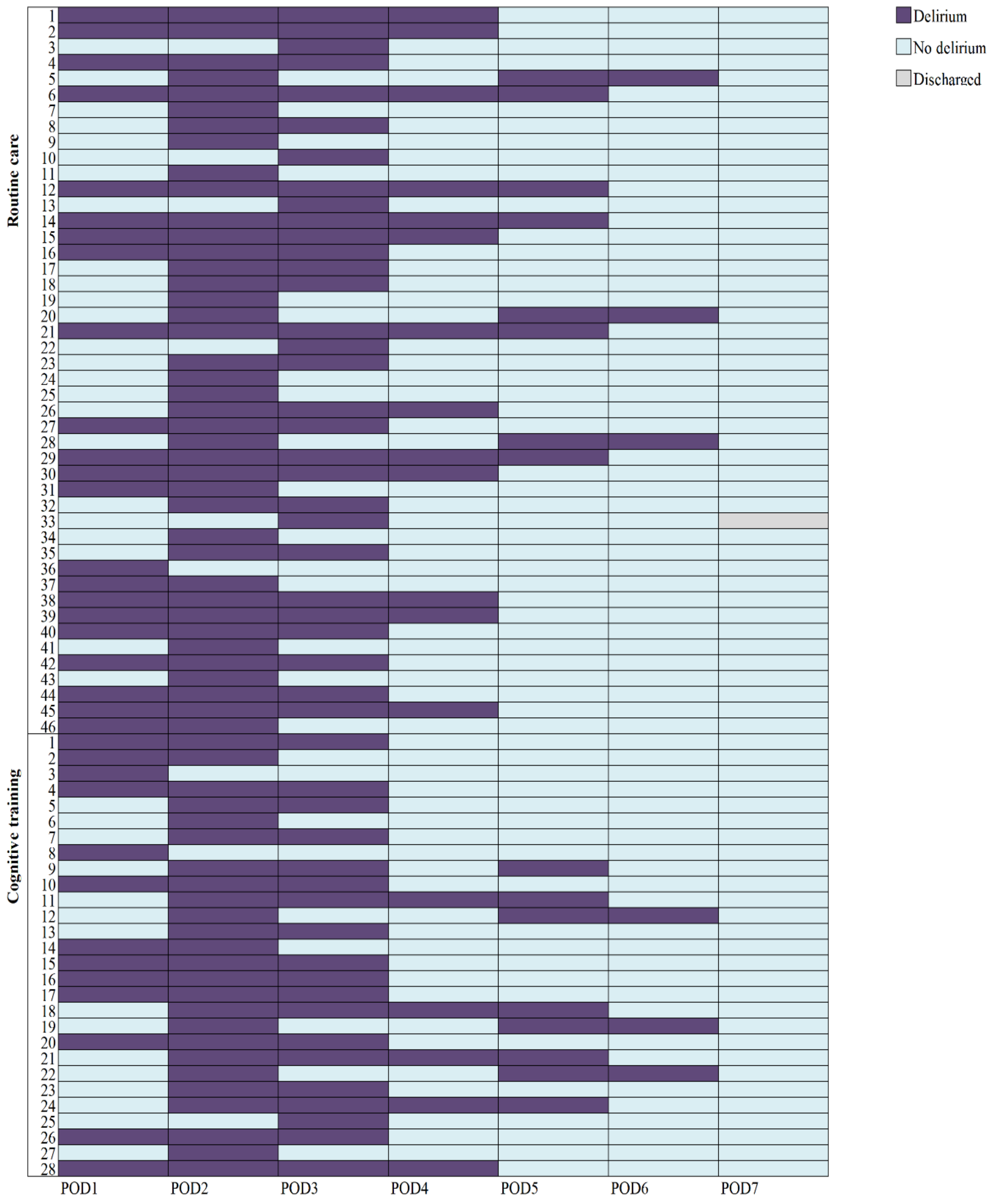

A total of 74 patients (28 patients assigned to cognitive training and 46 assigned to routine care) diagnosed as delirium from postoperative days (PODs) 1 through 7.

© 2024 Jiang Y et al. *JAMA Network Open.*

eFigure 4. MoCA Scores Over Time

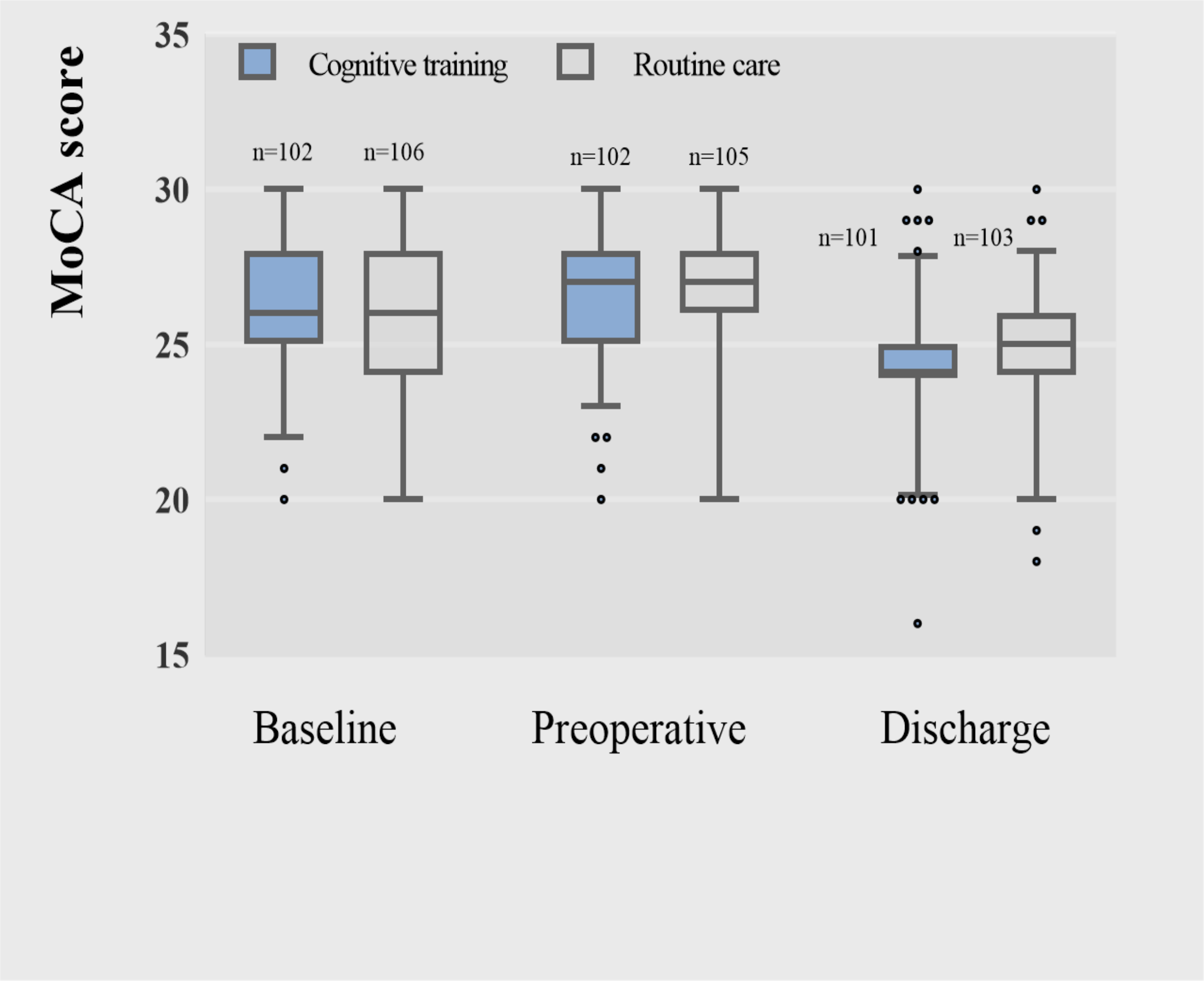

Abbreviations: MoCA, the Montreal Cognitive Assessment (range, 0 [worst] to 30 [best]).
The preoperative time point represents the day of surgery. The discharge time point represents postoperative day 7 or at discharge, whichever occurred first.

© 2024 Jiang Y et al. *JAMA Network Open.*

eTable 6. Potential Association Between the Total Hours of Cognitive Training and the Risk of Delirium

Duration of training，hour	OR (95%CI)	*P* Value	*P* for trend
1-2	1 [Reference]	NA	< 0.001
3	0.89 (0.30-2.63)	0.83	
4	0.33 (0.02-5.33)	0.44	
5	0.36 (0.05-2.60)	0.31	
6	0.43 (0.08-2.44)	0.34	
7	0.21 (0.03-1.45)	0.11	
8	0.14 (0.01-3.93)	0.25	
9	0.20 (0.01-5.87)	0.35	

Abbreviations: CI, confidence interval; NA, not applicable; OR, odds ratio

In the patients assigned to cognitive training, a decreasing trend showed a statistically significant potential dose-response association between the total hours of cognitive training and the incidence of postoperative delirium (Cuzick test, $P < 0.00$)

© 2024 Jiang Y et al. *JAMA Network Open.*

eFigure 5. Association of the Duration of Cognitive Training and the Risk of Delirium

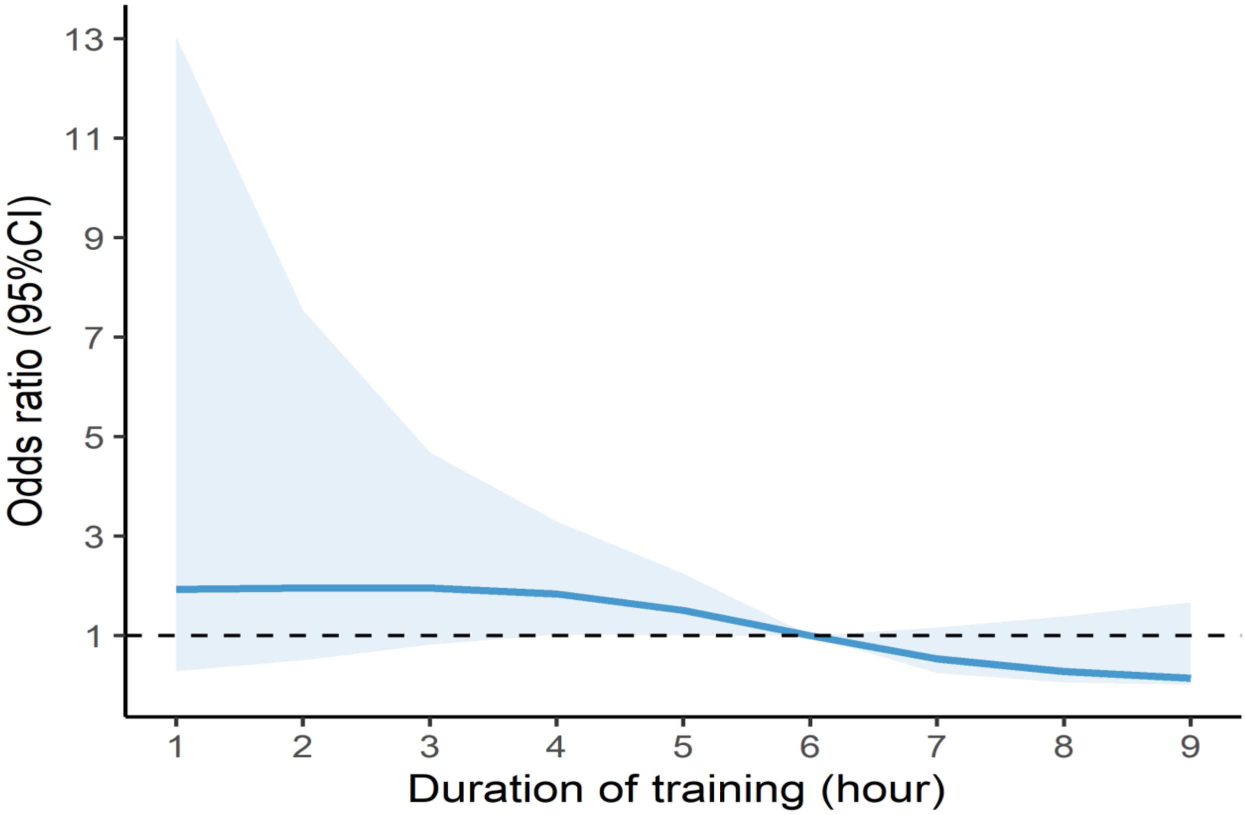

Abbreviations: h, hours.

There were 28 patients assigned to cognitive training who developed delirium, only 2 patients participated in 1 hour of cognitive training before surgery.

© 2024 Jiang Y et al. *JAMA Network Open.*
