# Supplementary material for: Cognitive Training for Reduction of Delirium in Patients Undergoing Cardiac Surgery: A Randomized Clinical Trial
Source: JAMA Netw Open. 2024 Apr 23;7(4):e247361. doi: 10.1001/jamanetworkopen.2024.7361 (PMC11040409; doi:10.1001/jamanetworkopen.2024.7361)
Supplement: Supplement 3. — Nonauthor Collaborators [file jamanetwopen-e247361-s003.pdf]

Supplemental Online Content: Nonauthor Collaborators
*First name, last name, and suffix (if applicable) are required and will appear in PubMed.

*Group Name(s): CT-LIFE study group							
***First Name and Middle Initial(s)**	***Last Name**	***Suffix (eg, Jr, III)**	Academic Degrees	Institution	Location (city, state/province, country)	Role or Contribution, eg, chair, principal investigator	Group (if more than 1 Group listed in the byline) and/or Subgroup (eg, Steering Committee)
Chengxin	Zhang		MD	Department of Cardiac Surgery, the First Affiliated Hospital of Anhui Medical University	Hefei, Anhui, China	Participating site-principal investigator	
Zhuang	Liu		MD	Department of Cardiac Surgery, the First Affiliated Hospital of Anhui Medical University	Hefei, Anhui, China	Participating site-principal investigator	
Fuhua	Huang		MD	Department of Cardiac Surgery, Nanjing First Hospital	Nanjing, Jiangsu, China	Participating site-principal investigator	
Xin	Chen		MD	Department of Cardiac Surgery, Nanjing First Hospital	Nanjing, Jiangsu, China	Participating site-principal investigator	
Yiming	Li		MD	Department of Cardiac Surgery, The First Affiliated Hospital of University of Science and Technology of China	Hefei, Anhui, China	Participating site-principal investigator	
Yang	Wang		MD	Department of Cardiac Surgery, The First Affiliated Hospital of University of Science and Technology of China	Hefei, Anhui, China	Participating site-principal investigator	
Haibo	Wu		MD	Department of Cardiac Surgery, The First Affiliated Hospital of University of Science and Technology of China	Hefei, Anhui, China	Participating site-principal investigator	
Qinfeng	Wei		MD	Department of Anesthesiology, the First Affiliated Hospital of Anhui Medical University	Hefei, Anhui, China	Data analysis	
Yinguang	Fan		PHD	Anhui Medical University	Hefei, Anhui, China	Data analysis	
