# Supplementary material for: Cognitive Training for Reduction of Delirium in Patients Undergoing Cardiac Surgery: A Randomized Clinical Trial
Source: JAMA Netw Open. 2024 Apr 23;7(4):e247361. doi: 10.1001/jamanetworkopen.2024.7361 (PMC11040409; doi:10.1001/jamanetworkopen.2024.7361)
Supplement: Supplement 4. — Data Sharing Statement [file jamanetwopen-e247361-s004.pdf]

Data Sharing Statement

Jiang. Cognitive Training for Reduction of Delirium in Patients Undergoing Cardiac Surgery. *JAMA Netw Open*. Published April 23, 2024. doi:10.1001/jamanetworkopen.2024.7361

Data
Data available: Yes
Data types: Deidentified participant data
How to access data: Send request email to Professor Xuesheng Liu: liuxuesheng@ahmu.edu.cn
When available: With publication

Supporting Documents
Document types: None

Additional Information
Who can access the data: Researchers whose proposed use of the data has been approved
Types of analyses: for a specified purpose
Mechanisms of data availability: After approval of a proposal, and with a signed data access agreement
